# Sub‐hourly resolution quality control of rain‐gauge data significantly improves regional sub‐daily return level estimates

**DOI:** 10.1002/qj.4357

**Published:** 2022-09-09

**Authors:** Roberto Villalobos‐Herrera, Stephen Blenkinsop, Selma B. Guerreiro, Tess O'Hara, Hayley J. Fowler

**Affiliations:** ^1^ School of Engineering Newcastle University Newcastle upon Tyne UK; ^2^ School of Civil Engineering Universidad de Costa Rica, Ciudad Universitaria Rodrigo Facio San José Costa Rica

**Keywords:** extremes, flooding, observations, precipitation, quality control, rainfall

## Abstract

This research demonstrates how the use of high‐resolution rain‐gauge data for quality control (QC) significantly changes extreme rainfall estimates, with implications in scientific, meteorological and engineering applications. Current open QC algorithms only consider data at hourly or daily accumulations. Here we present the first open QC algorithm utilising sub‐hourly rain‐gauge data from official networks at a national, multi‐decade scale. We use data from 1,301 rain‐gauges in Great Britain (GB) to develop a threshold‐based methodology for sub‐hourly QC that can be used to complement existing, freely available hourly QC methods by developing an algorithm for sub‐hourly QC that uses monthly thresholds for 1 hr, 15 min and 1 min rainfall totals. We then evaluated the effect of combining these QC procedures on rainfall distributions using graphical and statistical methods, with an emphasis on extreme value analysis. We demonstrate that the additional information in sub‐hourly rainfall allows our new QC to remove spuriously large values undetected by existing methods which generate errors in extreme rainfall estimates. This results in statistically significant differences between extreme rainfall estimates for 15 min and 1 hr accumulations, with smaller differences found for 6 and 24 hr totals. We also find that extremes in the distributions of 15 min and 1 hr rainfall accumulations tend to grow more rapidly with return period than for longer accumulation periods. We observe similarities between the shape parameter populations for 15 min and 1 hr rainfall accumulations, suggesting that hourly records may be used to improve shape parameter estimates for extreme sub‐hourly rainfall in GB. Sub‐hourly QC moderates unrealistically large return level estimates for short‐duration rainfall, with beneficial impacts on data required for the design of urban drainage infrastructure and the validation of high‐resolution climate models.

## Graphical abstract

Using a new threshold‐based algorithm we demonstrate that sub‐hourly rainfall records can be used to better distinguish spurious rainfall compared to hourly quality control (HQC) methods. Using this new algorithm on a large UK dataset we found significant differences in the GEV shape parameter estimates that moderate regional extreme rainfall return level estimates after sub‐hourly quality control (SHQC) is applied. Using sub‐hourly data therefore improves extreme rainfall estimates used in engineering and scientific applications.

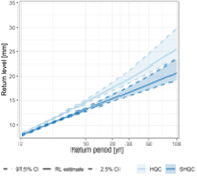

## 1 INTRODUCTION

This article introduces an extension to the hourly GSDR‐QC algorithm (Lewis *et al*., 2021), with additional quality‐control (QC) tests exploiting high‐resolution sub‐hourly rain‐gauge data. We demonstrate how sub‐hourly QC (SHQC) identifies suspect values that are obscured once data are aggregated to hourly (or longer) resolutions. We use extreme value analysis to demonstrate the added value of SHQC in the study of extreme rainfall, showing that it has significant effects on distribution parameters and regional return level estimates across a wide range of rainfall event accumulation periods. The methods used (GSDR‐QC and SHQC) are open source, customisable, and freely available (at https://github.com/nclwater/intense‐qc (Lewis *et al*., 2021) and https://github.com/nclwater/SubHourlyQC); they are intended for the *post hoc* QC of rainfall datasets which have not been subject to QC or which are compiled from multiple sources with different grades of QC, and can be utilised as an automated QC tool for any location where sub‐hourly or hourly (using GSDR‐QC only) rain‐gauge data are available, without need for corroborating radar or satellite observations. We encourage the use of these QC methods and welcome interaction with interested people.

The QC of weather observations is an essential prerequisite for the use of meteorological data (Estévez *et al*., 2011). In particular, the analysis of extremes, including their climatology and statistical properties, requires high confidence in the veracity of the extreme rainfall values present in rain‐gauge records (Blenkinsop *et al*., 2017). To this effect, the World Meteorological Organisation (WMO) publishes guidelines on the quality control of surface observations that recommend that data be subject to a series of tests to ensure their quality (WMO, 1986; 2021; Chambers *et al*., 2018).

However, procedures are not standardised across meteorological services and multiple sources of hydrometeorological data exist outside these institutions. In GB^1^ this includes the Environment Agency (EA) in England, Natural Resources Wales (NRW) and the Scottish Environment Protection Agency (SEPA). Data from these diverse sources are available to users with varying degrees of QC which does not meet WMO guidelines, and therefore, may require additional QC by users compared to that provided by institutions such as the UK Met Office (UKMO). However, data from these institutions underpin a significant amount of research (e.g. Jones *et al*., 2013; Dale *et al*., 2017; Darwish *et al*., 2021) and policy, including UK design rainfall methods (Kjeldsen *et al*., 2005; Stewart *et al*., 2013).

Meteorological services such as the UKMO and US National Oceanic and Atmospheric Administration (NOAA) maintain near‐real time QC procedures which are capable of identifying and correcting or removing errors on a daily or hourly basis (Kim *et al*., 2009; Qi *et al*., 2016; Met Office, 2020). However, their methods are not open and not well suited for the *post hoc* QC analyses carried out during the compilation of a new dataset which must be capable of identifying a wide range of errors potentially present in raw (pre‐QC) rain‐gauge records.

Systematic errors in rain‐gauge measurements include wind loss or under‐catch (Pollock *et al*., 2018), wetting loss, evaporation loss, and tipping errors (McMillan *et al*., 2012). The accumulation of these errors may result in 30% or higher underestimates of precipitation that reaches the ground, yet these errors are generally uncorrected. Tipping‐bucket rain‐gauges have additional sources of error due to their operating mechanism; they have a finite resolution (conventionally 0.2 mm) and the finite time taken by the bucket when it tips may cause appreciable errors for rainfall intensities over 250 mm·hr^−1^ (WMO, 2008). Rain‐gauge funnels are prone to obstructions, particularly by snow or vegetation. The removal of these blockages may produce sharp spikes in rainfall records (Wood *et al*., 2000; Rasmussen *et al*., 2012; Martinaitis *et al*., 2015).

Due to the varied nature of rain‐gauge measurement errors and the accumulation of errors over time (Morbidelli *et al*., 2017; 2018), the QC of large datasets compiled from multiple sources is a complex procedure best approached with automated algorithms. Durre *et al*. (2010) developed a set of 19 comprehensive, automated QC tests for daily observations of surface temperature, precipitation, snowfall and snow depth as part of the development of the Global Historical Climatology Network (GHCN) daily dataset. The Global Sub‐Daily Rainfall (GSDR) dataset was quality‐controlled using a set of 25 quality tests, covering the entire range of WMO‐recommended tests that are achievable relying solely on rainfall data, which flag possible faults in hourly rainfall records which are then evaluated by a rule‐base to remove suspicious data (Lewis *et al*., 2019; 2021). These tests built upon previous work which quality‐controlled a large hourly rainfall dataset for the UK using 11 single‐site tests (Blenkinsop *et al*., 2017) which were expanded with four tests against neighbouring gauges to generate a gridded hourly rainfall dataset for the UK (Lewis *et al*., 2018).

Sub‐hourly data allow for greater detail when examining rainfall time series, as variations in rainfall intensity and periods of suspiciously high tipping‐rates can be identified (Upton and Rahimi, 2003). However, the increased measurement variability and reduced spatial coherence in sub‐hourly data create additional challenges for QC. Sub‐hourly records tend to have shorter lengths than daily or hourly records, which may limit their usefulness for applications such as trend analysis and determining the climatology of extremes. QC algorithms which leverage sub‐hourly rain‐gauge data are rare in the scientific literature; they include a fully automated algorithm developed and tested on a short (2‐year) citizen‐science dataset (de Vos *et al*., 2019), and a semi‐automated QC algorithm developed for near‐real time use in two state‐wide and city‐wide meteorological stations in Oklahoma and Oklahoma City (McPherson *et al*., 2007; Basara *et al*., 2011). Note that it is feasible that other network operators use sub‐hourly data within their internal QC process, in automatic or semi‐automatic fashion, without publishing their methods in scientific journals.

Rain‐gauge datasets available at sub‐hourly resolution may not be subject to the same level of QC as those available at lower resolutions. In GB the sub‐hourly observations which form the basis of quality‐controlled hourly datasets (e.g. Lewis *et al*., 2018; Met Office, 2020a) are provided with limited or no QC. In Germany, 1 and 10 min precipitation data are freely available from the German weather service, Deutscher Wetterdienst (DWD), which also provides hourly data (DWD, 2019); however, they warn that “the quality of the high‐resolution observations cannot be as high as the quality of the hourly etc. aggregated data, because the latter are checked more thoroughly” (DWD, 2017). Overall, these differences in QC create barriers to the use of sub‐hourly data as their quality is perceived as inferior to hourly or daily products.

Sub‐hourly rainfall data are an important input for flood estimation and modelling, especially in urban settings or in small catchments sensitive to changes in rainfall intensity, with periods of high intensity leading to surface water flooding (Archer and Fowler, 2015; Ochoa‐Rodriguez *et al*., 2015; Cristiano *et al*., 2017; Peleg *et al*., 2017; Fadhel *et al*., 2018; Zhu *et al*., 2018). Flood estimation is important not only for civil engineering design but also for flood risk studies, vulnerability analyses, and emergency planning. Design rainfall estimates in GB, codified in the Flood Estimation Handbook (FEH) and available through the FEH Web Service (UKCEH (UK Centre for Ecology and Hydrology) and Wallingford HydroSolutions, 2015), are currently based on hourly data (Stewart *et al*., 2013). This leaves a gap where the statistical properties and frequencies of sub‐hourly extreme events in GB are not well characterised despite their importance for surface flooding in urban areas.

In this article we first describe the data and methods used to develop and test SHQC on a dataset of 1,301 rain‐gauges, producing what to our knowledge is the largest QC'd rain‐gauge dataset with sub‐hourly resolution in GB. We discuss the impact of QC, and the data resolution used by QC, on the general characteristics of the dataset before focusing our analysis on the changes QC has on rainfall extremes. We demonstrate that the targeted removal of suspicious extreme values has significant impacts on return level estimates and finally discuss implications of our work for engineering and scientific applications.

## 2 DATA AND METHODS

### 2.1 Data

Rain‐gauge measurements were obtained from three network operators within GB: the EA, NRW and SEPA. Portions of these rain‐gauge records have previously formed part of hourly datasets for the UK (Blenkinsop *et al*., 2017; Lewis *et al*., 2018), and of the GSDR dataset (Lewis *et al*., 2019). These data were updated to mid‐2018; however, only rain‐gauges with sub‐hourly temporal resolution have been used here to create what, to our knowledge, is the largest sub‐hourly rainfall dataset for GB. The inclusion of multiple providers has resulted in a mixture of tip‐time records and 15 min rainfall accumulations, and to a lesser degree, measurement resolutions. All data were considered at their original resolution during sub‐hourly quality control (SHQC).

**FIGURE 1:**
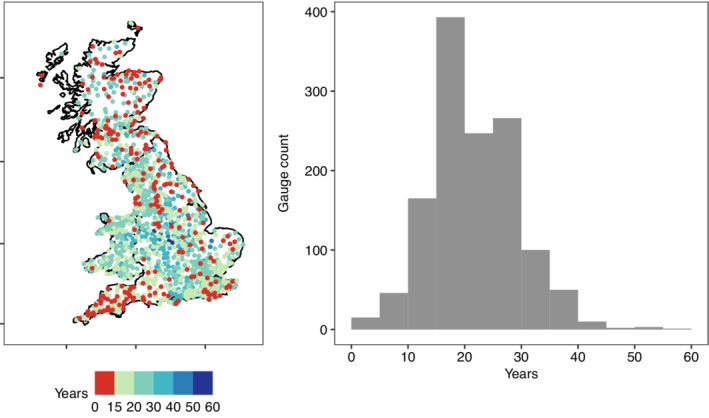
Map (left) and histogram (right) of sub‐hourly rain‐gauge record length, in years. Rain‐gauges with <15 complete years are highlighted in red on the map [Colour figure can be viewed at wileyonlinelibrary.com]

A total of 1,301 rain‐gauges with sub‐hourly data were identified. Their geographic distribution and a histogram of their record lengths prior to QC can be seen in Figure 1. Variations in gauge density and record length are evident, with the highest density in southeast England and relatively sparse coverage in the Scottish Highlands. Rain‐gauges with 30‐year or longer records are concentrated in England and western Wales, whilst Cornwall and Devon only have records under 20 years in length as the gauge network here is of more recent installation. The record length histogram shows that the bulk of the raw, pre‐QC rain‐gauges have between 10 and 30 years of data, with median record length at 20.5 years. In total the initial dataset contains approximately 27,600 station years of record.

QC information was not available for NRW and SEPA data beyond missing data flags. EA QC flags have significant temporal and regional variations, with close to 50% of the EA data between 2003 and 2008 flagged as “unchecked”; these flags have also failed to flag >300 unrealistically large totals (Blenkinsop *et al*., 2017). The absence of QC data for a large part of the data and the variations in QC application within the EA highlight the need for additional QC by users of these data and limit their usefulness; consequently, EA flags were not used in our QC process.

### 2.2 Quality‐control method

Two automated QC procedures were used on the rainfall data. The first QC stage was carried out using a modified version of the GDSR‐QC algorithm in Lewis *et al*. (2021) on hourly totals calculated from the raw sub‐hourly data; the modifications are detailed in the Supplementary Information. To distinguish the modified algorithm from the GSDR‐QC algorithm, and to highlight that it operates on hourly data, only the first stage of QC is referred to as *hourly quality control* (HQC). The latest version of the GSDR‐QC algorithm may be obtained at https://github.com/nclwater/intense‐qc, or https://pypi.org/project/intense/ (Lewis *et al*., 2021). The second QC algorithm operates using sub‐hourly data, so it is referred to as *sub‐hourly quality control* (SHQC). Within it we first ensure rainfall data have the correct frequency and resolution (SHQC/FR) before using a multi‐stage threshold approach (SHQC/T) to identify suspicious rainfall periods. The SHQC Python scripts are available at https://github.com/nclwater/SubHourlyQC.

The raw sub‐hourly data with tip‐time or 15 min resolution were aggregated to hourly resolution prior to HQC. The aggregation period consists of the 60 min prior to the hour, therefore the rainfall accumulated for 08:00 is the total rainfall registered from 07:00:01 to 08:00:00. This aggregation differs from that used by the UKMO in its Met Office Integrated Data Archive System (MIDAS), which records hourly totals with a 10 min offset (Met Office, 2020). Tipping‐bucket rain‐gauge records are temporally discontinuous as they register tip‐times and omit zero‐rainfall periods. Therefore, the aggregation procedure constructed a continuous time series by adding zero values between hours where precipitation was registered unless a missing flag data was present. In these cases, the gaps between non‐zero observations were padded with missing data values (“NA”). The hours marked as suspicious by the HQC results were replaced with “NA” values in the original sub‐hourly rainfall records, generating a baseline quality‐controlled sub‐hourly dataset which was then refined through SHQC.

A visual examination of seasonal rainfall extremes present in the sub‐hourly dataset after HQC highlighted suspicious events. For example, Figure S1 shows multiple 15 min totals exceeding 50 mm in magnitude present in Wales and Lincolnshire during autumn (SON). For reference, 50.8 mm over a 2.5 hour period was sufficient to cause significant surface flooding in Newcastle (Environment Agency, 2012); therefore, observing this volume of rainfall over 15 min would be highly remarkable and merits investigation. The SHQC algorithm was developed to identify and remove suspicious rainfall measurements that may not be remarkable or suspicious at 1 hr resolution.

The complete QC process was staged. First, the raw sub‐hourly rain‐gauge datasets were aggregated to hourly resolution and subject to HQC (see Supplementary Information for an overview, and Lewis *et al*. (2021) for additional details). The resulting QC'd hourly data were then disaggregated to their original sub‐hourly interval before being subject to SHQC (sections 2.2.2 and 2.2.3). Data gaps created by HQC were treated as missing data. In section 2.2.1 we describe the methods used to evaluate QC performance. The development and implementation of the SHQC procedure is shown before presenting the extreme value methods used to study the impact of SHQC on rainfall frequency estimation.

#### 2.2.1 QC diagnostic tools

The effect of SHQC was evaluated using frequency distribution plots for hourly rainfall, the annual maximum series (AMS) of sub‐hourly and hourly rainfall durations, and extreme value theory. Frequency distribution plots are a graphical representation of the entire frequency distribution of rainfall, which makes them useful for identifying abnormally frequent data. The extreme value analyses, carried out to establish the effect of SHQC on return level estimates, are described in detail in section 2.3.

The frequency distribution of rainfall was examined by calculating the relative frequency of non‐zero rainfall hours within discrete hourly rainfall bins; the relative contribution of rainfall within each bin to total rainfall was also calculated. Bin edges, $\;b_{i}$, are defined by:

(1) $$b_{i}=e^{\left\{\ln\left(a\right)+{\left[\frac{i}{n-1}{\left(\ln\left(c\right)-\ln\left(a\right)\right)}^{2}\right]}^{\frac{1}{2}}\right\}},$$

where a and c are the lower and upper limits of the range to be partitioned into n number of bins. This function is modified from a function presented in Klingaman *et al*. (2017) for use with daily precipitation climate model output. We use two (a,c,n) sets of parameters: (0.2, 100, 90) when examining the complete hourly rainfall distribution, and (0.2, 50, 50) when examining different SHQC threshold iterations. These parameters and function have been chosen because most hourly rainfall totals are low, therefore narrower bins are useful at the low end of the range, with wider bins to characterise less frequent heavy hourly rainfall. The bin edges generated by Equation (1) were rounded to the nearest 0.2 mm to account for the minimum resolution of most tipping‐bucket rain‐gauges, and finally 0.0 and ∞ were added as the smallest and largest bin edges, respectively.

The frequency distributions, i.e. the number of rainfall hours in each bin relative to the total number of rainfall hours, and the relative contributions to total rainfall, i.e. the total rainfall in each bin relative to total rainfall across all bins, were calculated at each gauge, for each month, and at each QC stage (raw data, after HQC, and after SHQC). Individual gauge relative frequency and rainfall contributions results were aggregated across the entire dataset on a monthly basis and plotted using log–log and semi‐log scales, respectively. These plots serve to examine abnormalities in rainfall distributions which signal the presence of suspicious data. Ideally a smooth decrease in rainfall event frequency should occur as rainfall magnitude increases, reflecting the rarity of large and extreme events in observations.

Two sets of reference events were used to evaluate the performance of SHQC. A training set was selected based on the preliminary examination of rainfall extremes carried out after HQC. All the hourly seasonal maximum precipitation events over 40 mm were selected, for a total of 68 events with a nearly uniform seasonal distribution: 15 events were present from each season, except for summer with 23 events. Each event was manually quality‐controlled: first, 3‐hour totals for each gauge and up to 10 of its closest neighbours (by distance) were calculated and compared. Then the sub‐hourly data for each of these events were plotted and inspected for spiking or indications of obstructions at hourly, 15 min and, when possible, 1 min temporal resolutions. Inter‐tip times were calculated to identify fast‐tipping problems in gauges with tip‐time data. Finally, the UKMO daily weather reports (Met Office, 2020b) were examined if doubts about the event's validity remained. All the selected winter (DJF) and spring (MAM) events were deemed suspicious, nine of 23 and two of 15 events were considered plausible in summer (JJA) and autumn (SON), respectively. The presence of suspicious events after HQC reflects the difficulty of quality‐controlling a large dataset where a balance must be struck between removing suspicious events whilst also retaining observed extremes and highlighted the need for further QC across the dataset. This dataset and the 1 and 15 min totals calculated for each event were the start of the SHQC threshold development.

A set of 30 validation events (listed in Table S2) were used after development of SHQC was completed to ensure that the rainfall associated with observed surface flooding events was preserved throughout the QC process. These events were recent examples of surface water flooding events driven primarily by extreme rainfall, compiled from multiple sources such as flood investigation reports prepared by local councils as part of their duties under section 19 (1) of the *Flood and Water Management Act 2010* or by the EA as part of ongoing work for the UK Department for Environment, Food & Rural Affairs' (DEFRA) Surface Water Management Action Plan, Boosting Action in Surface Water—Workstream B—Plausible Extremes in Surface Water (DEFRA, 2018). Rainfall data for up to 10 gauges within a 30 km radius of each surface flooding event were extracted for a 5‐day window extending 3 days prior and 1 day past the date on which flooding occurred; the highest rainfall totals for durations between 15 min and 96 hr were calculated and compared prior to and after SHQC to establish if these verified extreme events were removed or not by the QC.

#### 2.2.2 Sub‐hourly QC algorithm

Frequency checks (SHQC/FR) are required as the raw sub‐hourly datasets contained a mixture of different reporting frequencies, ranging from daily to tip‐times. To account for this, the modal data frequency for each month was calculated for each rain‐gauge; rainfall in those months that had reporting frequencies above 15 min was replaced with NA values. In addition, gauges with extended periods of rainfall measurements with integer (1 mm) resolution were identified; these are possibly the result of data processing or transmission problems where tip numbers were stored without converting to mm values, although metadata were unavailable to confirm this. As with the frequency checks, data resolution checks removing periods with integer rainfall measurements were carried out at monthly timeframes. SHQC/FR checks ensure that the rainfall data retained for analysis are of high temporal and depth resolutions.

The second step of SHQC consists of threshold checks (SHQC/T) that were developed iteratively: threshold values were selected, evaluated using the tools described in Section 2.2.1 and the set of 68 training events, and modified until a satisfactory result was obtained. The additional information provided by sub‐hourly records was used in two different ways during the SHQC/T process, reflecting the two different types of data available: tip‐time records and 15 min totals. Tip‐time records allow for a more thorough analysis of rainfall and its intensity, whilst 15 min records offer limited opportunities for analysis beyond the threshold tests. Therefore, an inter‐tip time test was implemented for use in gauges where tip‐time records were available, whilst threshold‐based tests were implemented for use in gauges without tip‐times.

Thresholds for suspicious data were selected for rainfall totals over three accumulation periods: 1 min, 15 min and 1 hr. The 15 min period represents the native resolution for ∼38% of rain‐gauges, whilst 1 min totals are used whenever tip‐times are available. Threshold selection is described in section 2.2.3.

The final SHQC/T algorithm tests rainfall data with monthly thresholds at increasing temporal resolution. The algorithm operates on a gauge‐by‐gauge basis. First, clock hours with rainfall totals that exceed the hourly rainfall threshold (TH_60,m_) are extracted for every month *m* and added to a pool of suspect hourly totals. The sub‐hourly rainfall record for the 3‐hour period (or “event”) centred on each suspect hour is extracted and the measurement frequency (F_data_) of the data is calculated. Two indicators are calculated for all 3‐hour periods: the number of large 15 min rainfall totals (N_15_) as well as the mean intensity of wet 15 min totals (${\bar{I}}_{15}$). Two additional indicators are calculated for events that have tip‐time data (i.e. F_data_ ≠ 15 min) available: the number of large 1 min totals (N_1_) and the most frequent inter‐tip time (T_mode_). Large 1 or 15 min totals are those that exceed the corresponding monthly thresholds for each month (TH_1,m_ and TH_15,m_ respectively). Warm month (MJJASO) events flagged by the hourly threshold that have either N_15_ > 1, or N_15_ = 1 and ${\bar{I}}_{15}$ > TH_15,m_ are removed (as with HQC, suspicious events have their data replaced with NA values in the time series); for periods flagged during cool months N_15_ > 0 is sufficient to justify their removal. The more relaxed condition for warmer months, which requires more than one large 15 min total, is intended to account for the higher probability of intense, convective showers during warm conditions. This was added as a result of the validation process, which found that the strict criterion used in cold months removed plausible summer events in 15 min gauges. The additional indicators for events with tip‐time data are used to remove events if very fast tipping occurs (T_mode_ < 2 seconds), or N_min_ > 0. Metadata for all examined events are saved in a log file so users can examine the SHQC/T results and identify any patterns amongst the removed events.

**FIGURE 2:**
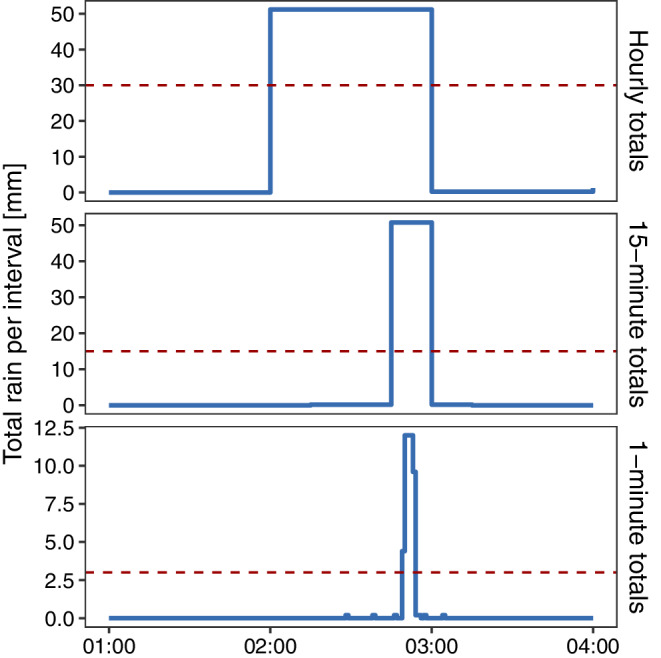
Example of abnormally large rainfall in gauge EA589294, Seathwaite Tarn, on 27 January 2013 for different accumulation periods. The final thresholds used by SHQC/T for January at each accumulation period are shown with a horizontal dashed line [Colour figure can be viewed at wileyonlinelibrary.com]

An example of how the final SHQC/T algorithm can use the additional information available in sub‐hourly rainfall records is illustrated with the large hourly total shown in Figure 2, which was recorded by an EA gauge located in northwestern England. This event has an hourly total of 51.2 mm, well below the GB record of 92 mm and was not removed by the HQC algorithm. The same 3 hr period is shown with increasing temporal resolutions matching those used by the SHQC/T procedure. First a large hourly total is detected for 03:00. The data frequency check correctly identified tip‐time data, and the inter‐tip time check was carried out. T_mode_ was found to be 1 s; this is sufficient to flag the 3 hr period as suspicious and remove it by replacing the original values with NA in the time series. In addition, the 1 and 15 min thresholds are exceeded, with N_1_ = 4 and N_15_ = 1, confirming the period as suspicious. Posterior checks of the original EA metadata provide increased confidence in the algorithm's result as the EA flag for this period is “S” (suspect), and snow was noted to have affected measurements between 28 December 2012 and 14 February 2013. Snowmelt events are a frequent source of fast‐tip errors in rain‐gauge data (Upton and Rahimi, 2003). The very fast tips detected by SHQC/T are consistent with a snowmelt event and represent information which is not available when examining hourly data. Other causes of fast‐tipping events logged in rain‐gauge metadata include manual tampering, electrical faults and vegetation blockages which when released can cause erroneous measurements (Wood *et al*., 2000). The impact of removing this and other large hourly totals from this gauge's record is explored in further detail in section 3.

#### 2.2.3 SHQC/T threshold selection

**FIGURE 3:**
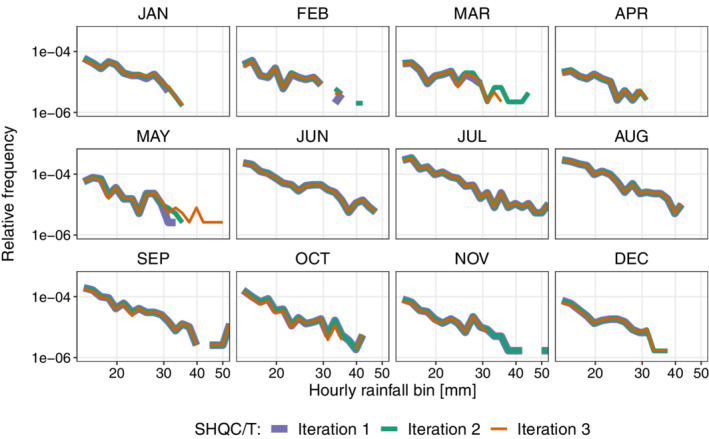
Log–log plot of relative frequency of hourly rainfall events over 10 mm after three different SHQC/T iterations, indicated with different line types and colours. Relative frequency is calculated by dividing the number of hours in each rainfall bin, $N_{\mathrm{bin}},$ by the total amount of wet hours, $N_{\text{total}},$ registered each month; these sums are shown here for the subset of gauges impacted by SHQC/T [Colour figure can be viewed at wileyonlinelibrary.com]

**FIGURE 4:**
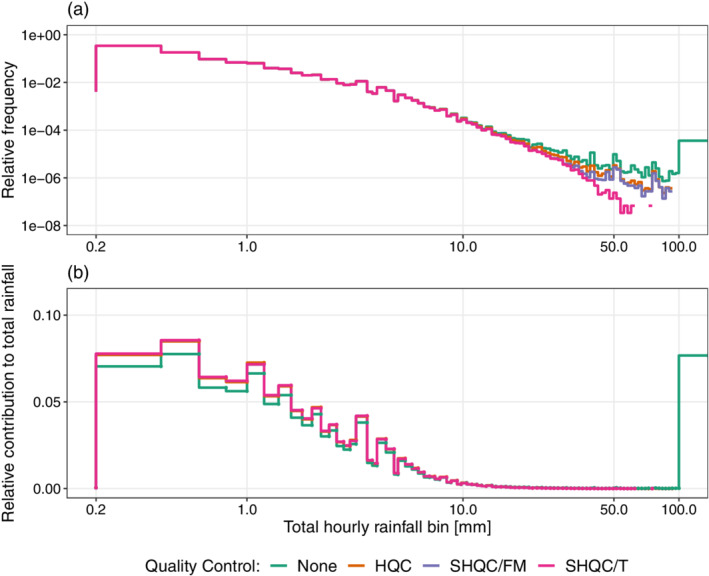
Relative frequency of (a) rainfall and (b) contribution to total rainfall, of hourly rainfall bins before QC (green), after HQC (red), after SHQC/FR (purple) and after SHQC/T (pink). Relative frequency is calculated by dividing the number of hours in each rainfall bin, $N_{\mathrm{bin}},$ by the total amount of wet hours, $N_{\text{total}},$ recorded in the entire dataset [Colour figure can be viewed at wileyonlinelibrary.com]

Three iterations were required to obtain a satisfactory set of thresholds for the SHQC/T procedure. The presence of large outliers in the seasonal maximum plots (e.g. Figure S1) was checked at each iteration, as well as distribution smoothness in the relative frequency plots (Figures 3 and 4), and good performance in our training and validation set of events.

The initial set of thresholds were chosen to allow the algorithm to replicate the manual quality‐control classification done on the training set of events, and all iterations were validated to ensure they correctly identified the manually identified erroneous events. Values were initially defined for meteorological seasons to reflect seasonal variations in the drivers of precipitation in GB (Chandler and Gregory, 1976; Hulme and Barrow, 1997); winter (DJF) and spring (MAM) were considered as cool weather seasons when intense convection is less likely to drive high sub‐hourly extremes, whilst summer (JJA) and autumn (SON) were grouped into warm weather months. Cool months had initial hourly, 15 and 1 min thresholds of 30, 10 and 2 mm, respectively, with higher 40, 20 and 5 mm thresholds set for the same rainfall accumulation periods during warm months.

These combinations yielded encouraging results for JJAS, which retained the same thresholds throughout the iteration process. Figure 3 illustrates the effect that different SHQC/T parameter iterations had on the upper end of the rainfall distributions: iteration one results show a very sharp drop‐off in rainfall events in the rainfall distribution plots for winter and spring months (e.g. December and May); crucially, manual checks of some spring events revealed plausible events had been removed. This motivated a second iteration where the 15 and 1 min thresholds for winter were increased to 15 and 4 mm, respectively. However, these increases allowed events flagged as suspicious in the training set to remain after SHQC/T during spring (e.g. February and March, Figure 3), as events in the training set that had been discarded correctly by the initial thresholds were not removed by the higher thresholds of the second iteration.

The final 15 and 1 min thresholds (Table 1) avoid the sharp cut‐off in event frequency seen in iteration 1 and correctly remove suspicious events in the training set that iteration 2 had not removed. The thresholds in iteration 3 discarded the seasonal approach in favour of monthly thresholds that have a smoother transition between the high‐threshold values of JJAS and the low thresholds in FMA, reflecting the annual GB cycle of increased and decreased convective rainfall (Hulme and Barrow, 1997). Thresholds decrease at a slow rate during ONDJ as autumn and winter storms, fuelled by relatively warm ocean waters, can still yield significant rainfall in short periods of time. The rapid increase between AMJ reflects an increased possibility of convection in May as days lengthen and warm up; the effect of these threshold changes is clearly seen in the smoother tail of extreme events in the May panel of Figure 3.

**TABLE 1:** Monthly thresholds (in mm) for SHQC/T

Rainfall accumulation period	Month (Dec–Nov)											
	D	J	F	M	A	M	J	J	A	S	O	N
1 hr	30	30	30	30	30	40	40	40	40	40	30	30
15 min	15	15	13	13	13	18	20	20	20	20	17	16
1 min	3	3	2	2	2	4	5	5	5	5	4	3

There are small changes to the total number of events removed during the iteration process (Figure S2). The number of events that were not removed by SHQC/T increased between iterations 1 and 2 as fewer events with tip‐time/15 min data were removed and the number of fast‐tipping events remained constant. Changes to hourly thresholds from iteration 2 to iteration 3 increased the number of events evaluated by SHQC/T, which had remained constant between the first iterations. The timing of the events being removed also changed, with fewer events removed in May and more events removed from October and November, in line with the changed thresholds.

### 2.3 Extreme value analysis

The net effect of QC on extreme rainfall analysis, one of the target applications, was tested using the generalised extreme value (GEV) family of distributions. The GEV is used to model the distribution of extreme values of a series of independent observations $X_{1},X_{2},\ldots ,X_{n}$ which are blocked into sequences of length n; here we use 1 year as n. The maximum values of m blocks of observations generate the AMS series $M_{n,1},\ldots ,M_{n,m}$, to which the GEV can be fitted. The GEV is defined as:

(2) $$\mathrm{GEV}\left(\mu ,\sigma ,\xi \right)=\exp\left\{-{\left[1+\xi \left(\frac{M_{i}-\mu }{\sigma }\right)\right]}^{-1/\xi }\right\},$$

where μ is the location parameter, σ is the scale parameter and ξ is the shape parameter. The value of ξ determines the behaviour of the tail (extreme large values) and is used to classify the GEV into three families. The Gumbel distribution family corresponds to the subset of the GEV family with ξ=0 and is described as light tailed, with a linear return level plot (a plot of return level estimates against the logarithm of return period); if ξ>0 the return level plot will be concave and lack a finite bound, this behaviour is described as heavy tailed and the distribution belongs to the Fréchet family; finally if ξ<0 the distribution belongs to the Weibull family which is upper bounded with a convex return level plot (Coles, 2001).

The GEV was fitted to the single‐gauge AMS extracted from sub‐hourly data recalculated to 15 min, 1, 3, 6 and 24 hr accumulation periods. Years with >15% missing data were excluded and a minimum of 15 years with acceptably complete data was required at each gauge; this decreased the number of gauges available for analysis to 897 (Figure 1). Whilst longer records allow for more robust estimations, geographic coverage was prioritised; selecting gauges with longer records would have excluded Cornwall and Devon (southwest England) from the analysis. The fitted GEV was used to estimate events with 0.2 and 0.1 annual exceedance probabilities (AEP); these have average return periods of 5 and 10 years, respectively. These AEPs were chosen due to the shortness of records (median gauge record length is ∼20 years).

L‐moments were used to estimate the GEV parameters as they are well‐suited for use in small samples (Hosking, 1990). This method has been previously used for the analysis of a large UK hourly rainfall dataset (Darwish *et al*., 2018; 2021) as well as for global datasets. In particular an analysis by Papalexiou and Koutsoyiannis (2013) of 15,137 globally distributed daily rainfall records found that the GEV ξ parameter tended to 0.114 as record length increased. Graphical analyses are used to see if SHQC has a significant effect on ξ due to its importance to the behaviour of extremes.

The effect of QC on rare event estimates (up to 1% AEP – 100‐year return period) was evaluated using an index flood type of regional frequency analysis (Hosking and Wallis, 1997; Faulkner, 1999; Reed *et al*., 1999; Stewart *et al*., 2013). As usual for this family of methods, the AMS of different rain‐gauges were standardised using their mean value and then pooled together. Regional L‐moment values were then calculated and used to fit a regional GEV distribution, including regional average GEV shape parameters that have the same interpretations to single‐gauge shape parameters.

Regions defined for all rain‐gauges contain the 14 closest rain‐gauges (according to Euclidean distance) in addition to the target rain‐gauge, for a total of 15 sites per region. Larger 25 rain‐gauge regions were also tested (results not shown) but the addition of more distant gauges was detrimental. The heterogeneity measure H (Hosking and Wallis, 1997) was used to evaluate the quality of each region: if H<1 a region is homogeneous, i.e. of good quality. The gauge discordancy measure *D* (Hosking and Wallis, 1997) serves as an indicator that a rain‐gauge is an outlier to the region where it is included. Gauges with D>3 are considered discordant for regions with 15 sites. Regional homogeneity and the number of discordant rain‐gauges were calculated for all regions before and after SHQC. Regional return‐level estimates and plots were compared before and after SHQC, with confidence intervals estimated through Monte Carlo simulation. All regional frequency analysis was carried out in R (R Development Core Team, 2006) using the lmomRFA package (Hosking, 2019). This regional frequency analysis is comparable to that used in the UK and elsewhere (Svensson and Jones, 2010) and serves to illustrate the impact of SHQC on the results from regional frequency analyses.

## 3 RESULTS AND DISCUSSION

### 3.1 Data removed by QC

The different QC stages remove different amounts of wet hours and total rainfall, with SHQC/FR removing the most wet hours and HQC removing the most rainfall (Table 2). This is expected as HQC is run first and has a greater number of quality checks compared to SHQC. The average intensity of rainfall removed by HQC and SHQC/T is higher than by SHQC/FR, reflecting the nature of each QC stage, where SHQC/FR removes data with coarse measurement resolution or frequency whilst HQC and SHQC/T remove suspicious extremes. SHQC/T checks examined 698 3‐hr periods from 227 gauges; 597 periods were deemed suspicious (85%) and removed. Rain‐gauges with 15 min and tip‐time resolutions are evenly represented amongst removed periods (52 and 48% respectively), and the proportion of events removed is very similar for both temporal resolutions (86 and 84% for 15 min and tip‐time data respectively).

**TABLE 2:** Summary of data removed by each QC stage. Percentages are relative to the pre‐QC data

QC	Wet hours	Rainfall [mm]	Average intensity of removed wet hours [mm·hr^−1^]
HQC	4.13 × 10^4^ (0.137%)	2.55 × 10^6^ (8.64%)	61.6
SHQC/FR	4.79 × 10^5^ (1.59%)	5.87 × 10^5^ (1.99%)	1.23
SHQC/T	902 (0.003%)	3.41 × 10^4^ (0.12%)	36.3

### 3.2 QC validation

The final thresholds used by SHQC/T were validated in three ways. First, the SHQC results were compared with the original manual QC of the training set of events. These were successfully classified into suspect or plausible events. Next, further manual QC was carried out for two sets of events to assess the probability of false positives (not removing suspect events) or false negatives (removing events which are plausible). To test for false positives, all events over 40 mm in an hour were manually checked; 14 out of 74 such events were considered suspicious, leading to a 19% false positive rate. To test for false negatives, all events removed by SHQC/T that had significant rainfall in nearby gauges (gauges within 30 km that had at least 50% of the suspect event's 6 hr total; the increased time window is used to allow for differences in rainfall timing) were subject to manual QC; this found that 7 out of 72 events were plausible, therefore the false negative rate of the sample was 10%.

Finally, the capacity of SHQC/T thresholds to preserve extremes associated with a reference set of 30 observed pluvial flooding events was tested. In total, 184 rain‐gauges were found to contain rainfall data relevant to 26 floods. Two events lie beyond the temporal domain present in the data and could not be evaluated, and no rainfall was registered at nearby rain‐gauges during flooding events in Bury St Edmunds, Suffolk, 19 September 2014 and Coverack, Cornwall, 23 August 2017 (Suffolk County Council, 2015; Cornwall Council and Environment Agency, 2018). Two of the pluvial flood events (Dawlish, Devon on 5 May 2012 and Bar Hill, Cambridge on 8 August 2014) had hourly rainfall totals large enough (exceeding 40 mm) to trigger the SHQC/T algorithm. The SHQC check found both events to be plausible and no data were removed. An examination of the 15 min and 1 hr rainfall totals associated with these pluvial flood events shows four instances where TH_15_ is exceeded or matched; however, none of these coincide with exceedances of TH_60_ and in all events N_15_ = 1 therefore they would not have been removed even if a lower TH_60_ had been used. These results show that the thresholds used by SHQC are sufficiently high to avoid removing flood‐generating rainfall. A summary of each event's results is shown in Table S2.

### 3.3 QC effects on rainfall distribution

The rainfall distribution plot shown in Figure 4a reveals the effect that HQC, SMQC/FR and SHQC/T have on the rainfall frequency, with remarkable impacts on extreme values. Before any QC is attempted, the dataset contains a relatively high frequency of very large events with hourly totals larger than 100 mm. These values deviate from a smoothly decaying trajectory and show an increase in frequency with event magnitude, something which violates the expected behaviour of rainfall extremes. Note that the sharp increase in event frequency in the final bin corresponds to all hourly totals over 100 mm, although this behaviour is robust and has been observed when using higher upper limits (up to 133 mm) in the binning function. The effect of HQC on the rainfall distribution is clear, with no remaining data over the UKMO‐accepted record of 92 mm in an hour (Met Office, 2020c); however, extreme hourly values continue to be relatively frequent. SHQC/FR has a relatively small impact on the distribution of hourly rainfall totals as no large deviations from the HQC data are observed. This suggests that the large number of hours removed by SHQC/FR followed largely the same distribution as the HQC data, including some extreme values. In contrast, SHQC/T further reduces the frequency of extreme hourly events, with a smoother decay in frequency as rainfall magnitude increases than was obtained after HQC. This is desirable as larger magnitude events should generally decay in frequency, especially for the large samples presented here.

Overall, total rainfall volume is dominated by the contribution of hours with <10 mm (Figure 4b); however, the raw dataset shows a very large contribution to total rainfall from the >100 mm hourly rainfall bin. This large contribution was traced to a single gauge (EAR50430_FW) which has 15 min totals in excess of 9,000 mm over a 3‐day period in November 2004 which were correctly identified as suspicious by HQC. There are small differences between the relative contributions of extreme rainfall to total rainfall for HQC, SHQC/FR and SHQC/T; therefore, a high‐quality hourly QC algorithm may be sufficient for rainfall data applications such as water resource management where accuracy in representing rainfall volume is of greater importance than representing extreme events.

### 3.4 SHQC effects on extreme rainfall

SHQC/T has the largest impact on the distribution of sub‐hourly extreme rainfall of any QC component considered here. This contrasts with the fact that HQC and SHQC/FR remove much larger amounts of hours and rainfall from the database (Figure 4a). This reflects the targeted nature of SHQC/T, which aims to distinguish between real and spurious sub‐hourly rainfall extremes, and the more general checks contained in HQC and SHQC/FR.

SHQC/FR and SHQC/T have different effects on extremes, with SHQC/FR causing shorter AMS as periods with undesirable frequency or resolution are removed whilst SHQC/T does not affect AMS length as it removes much shorter periods (Figure S4). Rain‐gauge records modified by SHQC/FR show strong clustering in southwest and central England, as well as in southern and the central belt of Scotland. This suggests that the mixture of hourly and sub‐hourly records or the storage of tip‐numbers rather than rainfall amounts are both systemic errors in data recording and storage procedures.

Changes due to SHQC/T in the rainfall totals present in a gauge's AMS (Figure S5) are caused by the removal of rainfall periods believed to be spurious based on their sub‐hourly properties. This targeted removal of plausibly erroneous extremes does not generally result in a shortening of a gauge's AMS (Figure S4). The large amounts of data removed by HQC (described in the Supplementary Information) result in shorter rain‐gauge records that contain extreme events of reduced magnitude compared to the records prior to QC (Figure S4 and S5). Whilst some clustering is seen in gauges modified by SHQC/T, there is less evidence of systematic errors. The presence of multiple gauges with errors in Wales, Cumbria and the Scottish Highlands could suggest snow blockages as a cause for some clustering; however, evidence of snow blockages has been found for gauges in southeast England. There is no strong seasonal pattern to the monthly count of events removed by SHQC/T; the April and November peaks in monthly events removed are caused by repeated errors in three gauges during these months (Figure S6).

### 3.5 Impact of SHQC on frequency analysis

In this section the analyses have been made considering the changes that SHQC/T has made to rain gauges' AMS. SHQC/FM changes to AMS have been excluded from the comparison between HQC and SHQC datasets as SHQC/FM handles data frequency and formatting limitations rather than erroneous values. All the changes presented in this section are therefore due to the removal of data deemed suspicious by SHQC/T.

#### 3.5.1 Regional heterogeneity and discordant gauges

**FIGURE 5:**
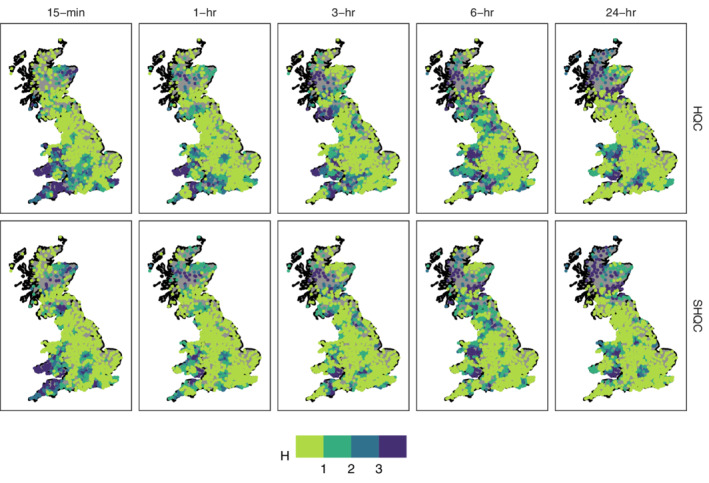
Heterogeneity measure of Hosking and Wallis (1997) as calculated for a 15‐rain‐gauge region centred around each rain‐gauge location. Green regions with *H* < 1 are considered homogeneous and of acceptable quality for regional frequency estimation [Colour figure can be viewed at wileyonlinelibrary.com]

The removal of erroneous, outlying values by SHQC/T improves the performance – as measured by regional heterogeneity and number of discordant gauges – of regional frequency analysis across all durations. The number of homogeneous regions increases by at least 10% after SHQC (Table 3), except at 24 hr, where a smaller 2% increase reflects the masking effect of data aggregation and the decreasing likelihood that a 24 hr annual maximum coincides with a value removed by SHQC. The overall increase in homogeneous regions after SHQC is not uniformly distributed (Figure 5); substantial improvements can be seen in Kent (all durations), south Wales (1–6 hr durations) and east of the Severn Estuary (all durations); a reduction in the homogeneity of some areas is also seen (e.g. southern Scotland, at 15 min duration). The number of regions with discordant gauges is also reduced after SHQC (Table 4); again, the effect is smaller at 24 hr than it is at shorter durations.

**TABLE 3:** Number of homogeneous regions (*H* < 1) after HQC and SHQC and the % change calculated as change (%) $=100\times \frac{\left(\text{SHQC}-\mathrm{HQC}\right)}{\mathrm{HQC}}$

Duration	HQC	SHQC	Change (%)
15 min	690	773	12
1 hr	784	861	10
3 hr	745	848	14
6 hr	627	731	17
24 hr	841	854	2

**TABLE 4:** Number of regions with discordant gauges in after HQC and SHQC

Discordant gauges in region	15 min		1 hr		3 hr		6 hr		24 hr	
	HQC	SHQC	HQC	SHQC	HQC	SHQC	HQC	SHQC	HQC	SHQC
0	913	1,000	844	937	887	976	860	928	907	892
1	341	262	406	327	364	284	390	301	355	376
2	30	22	34	20	31	23	34	55	21	15
3	NA	NA	NA	NA	2	1	NA	NA	1	1

#### 3.5.2 Impact of QC on GEV shape parameter

**FIGURE 6:**
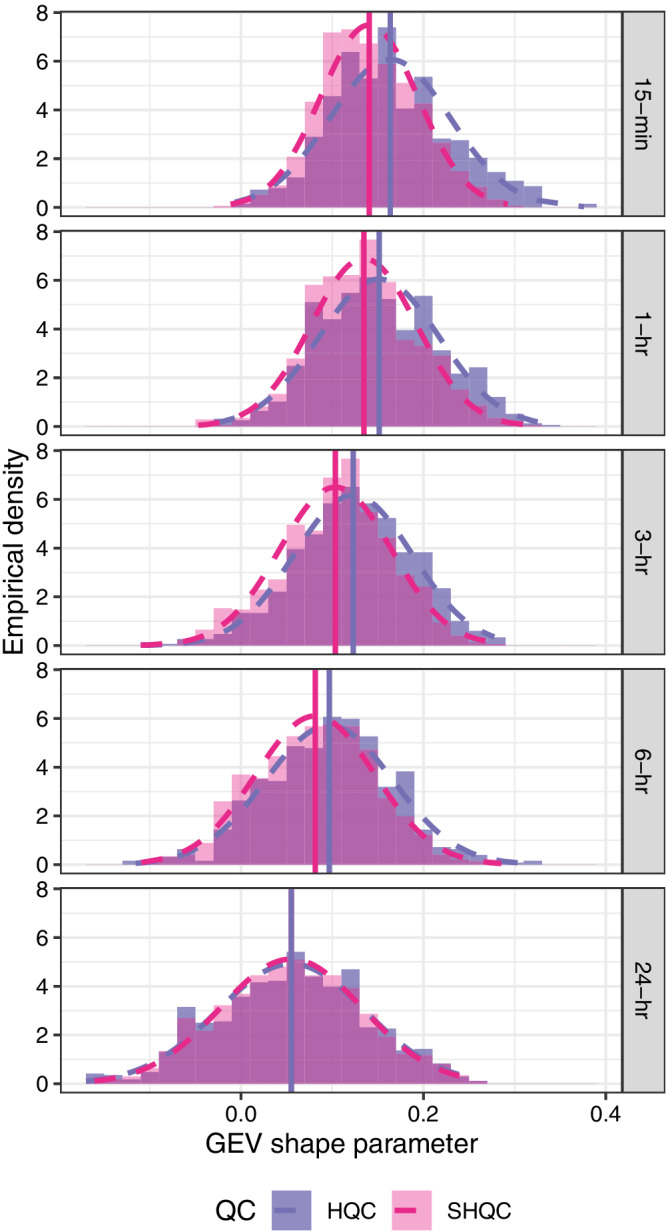
Empirical distributions of the regional GEV shape parameter for each duration, after HQC and SHQC – for regions with acceptable heterogeneity (*H* < 1). Fitted normal distributions are shown as dashed lines, and the mean values of each distribution are shown as vertical lines [Colour figure can be viewed at wileyonlinelibrary.com]

SHQC has a moderating effect on both single‐gauge (Figure S7) and regional (Figure 6) estimates of the GEV shape parameter (ξ). Focusing on the more robust regional estimates of ξ, and considering regions that are homogeneous, we can see that the distributions of ξ before and after SHQC are distinct for all durations under 24 hr. This is evident both in the empirical and fitted normal distributions shown in Figure 6. Mean ξ values are lower after SHQC (except at 24 hr where they are the same).

The distributions of single‐gauge estimates of ξ show strong similarities between 15 min and 1 hr durations, as well as between 6 and 24 hr durations (Figure S7 and Table S3). This effect is weaker in the regional estimates of ξ, with small similarities between 15 min and 1 hr duration, though the 6 and 24 hr ξ distributions are clearly different (Figure 6).

There is a trend towards lower mean ξ values as duration increases, from 0.14 at 15 min to 0.06 at 24 hr, both after SHQC. This contradicts results presented by Overeem *et al*. (2008) and Stephenson *et al*. (2016) who found no trend in ξ estimates across durations between 1 and 24 hr; the former studied a 12‐gauge region in the Netherlands, and the latter studied 182 rain‐gauges in New South Wales, Australia. This decreasing trend in ξ also contradicts conventional assumptions in the construction of intensity–duration and depth–duration frequency (IDF/DDF) models (Koutsoyiannis *et al*., 1998; Faulkner, 1999; Stewart *et al*., 2013).

The majority of ξ values for all durations are positive (especially at hourly and 15 min durations), indicating that a Fréchet distribution family is most frequent for extreme 15 min to 24 hr rainfall in GB, and implying that it is unlikely that an upper bound exists on the distributions of GB sub‐daily rainfall extremes. We find that the distribution of ξ at each duration is well approximated by the normal distribution (Figure 6), something that has been previously observed for daily rainfall at a global scale (Papalexiou and Koutsoyiannis, 2013), and which may be useful for simulation purposes.

### 3.6 Impact of QC on return level estimates

**FIGURE 7:**
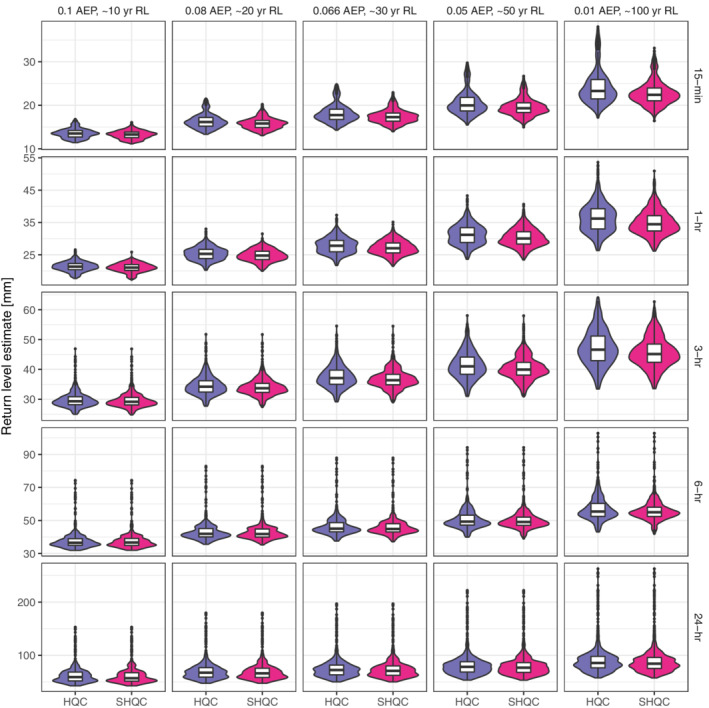
Return level estimate violin and box plots at different annual exceedance probabilities (top panel labels) and event accumulation periods (right panel labels), after HQC and SHQC for all regions with acceptable heterogeneity (*H* < 1). Violin plots are density plots, mirrored about the vertical axis and plotted using a kernel density estimate. The bold line in the boxplot inserts shows the median, the upper and lower edges of the box show the 75 and 25% quantiles respectively, whiskers extend up to 1.5 × the inter‐quartile range beyond the box edges [Colour figure can be viewed at wileyonlinelibrary.com]

**FIGURE 8:**
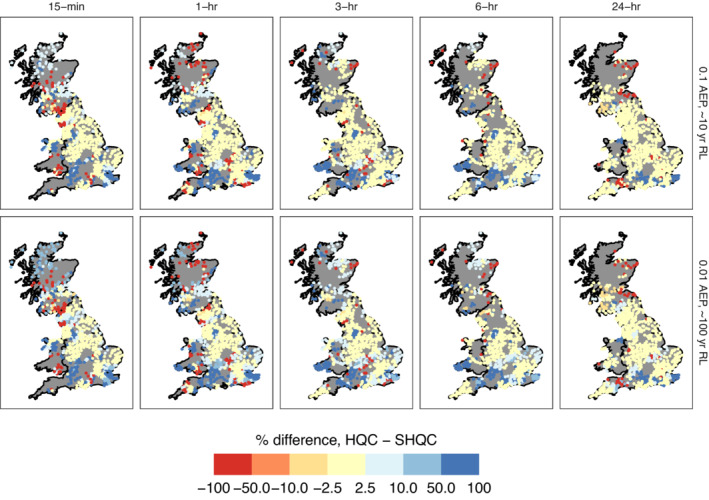
Percentage difference in regional return level estimates before and after SHQC, rainfall durations indicated on panel tops, AEP on panel right. Regions with +100% difference were heterogeneous prior to SHQC, and regions with −100% difference are heterogeneous after SHQC [Colour figure can be viewed at wileyonlinelibrary.com]

SHQC moderates the largest return level estimates of events of 3 hr or shorter, in both single‐gauge and regional analyses (Figure 7 and Figure S8), with changes more evident for higher return periods due to SHQC‐moderated ξ values which reduce growth curve steepness. SHQC has much smaller effects on 6 and 24 hr return level estimates. Overall differences in regional return level estimates are modest, with most regions showing differences of under ±2.5% (Figure 8), though changes in single regions can be substantial: for example, at Ashcombe, Devon, the regional 24 hr, 0.01 AEP estimates of 111.5 and 97.7 mm before and after SHQC differ by 14% (relative to the SHQC estimate). Differences at shorter durations can be larger, for example, at Dowdeswell, Cheltenham there is a 7.6 mm (29.5%) difference in the 0.01 AEP, 15 min return level estimates before and after SHQC (final value: 25.8 mm).

The merit of regional frequency analysis is clear, as single‐gauge estimates can have much larger differences due to SHQC. For example, the largest difference for a 24 hr event at 0.1 AEP at an individual gauge is of 122.1 mm, with an SHQC return level estimate of 107.6 mm. This 113% error is much larger than the mean event at this accumulation period (63.4 mm). Regional frequency estimates pool AMS from multiple gauges, reducing the influence that single‐gauge outliers have on return levels.

### 3.7 Return level estimate case‐studies

The impact of SHQC on single‐site return level estimates was evaluated using two locations: Seathwaite Tarn, located in Cumbria at an elevation of 378 m and in a sparsely populated catchment, and Temple Ewell in Kent, at an elevation 29 m and upstream of Dover. Both regions are prone to flash flooding, with multiple instances on record since 1700 (Archer and Fowler, 2021) and Temple Ewell sits upstream of Dover in the catchment of the River Dour, where intense rainfall has caused flooding (Dover District Council, 2019).

SHQC/T inspected five episodes at Seathwaite Tarn, of which four were removed due to fast‐tipping behaviour, including the 51.2 mm hourly total seen in Figure 2 and discussed in Section 2.2.3. These removals caused two changes to the 15 min AMS, with the values for 2013 and 2015 changing from 50.8 mm and 38.4 mm to 11.4 mm and 5.8 mm, respectively. At Temple Ewell four episodes were examined, with two removals due to fast tipping and one due to the presence of three values over TH_15,m_. Metadata did not provide causes for the errors, but the removed data were flagged as “S” by the EA and one episode on 20 December 2009 may be associated with snowmelt according to checks against the UKMO Daily Weather Summary for the date (Met Office, 2010). The 15 min AMS series was modified, with values from 2000 to 2009 changing from 16.4 and 41.6 mm to 15.6 and 13.4 mm, respectively.

**FIGURE 9:**
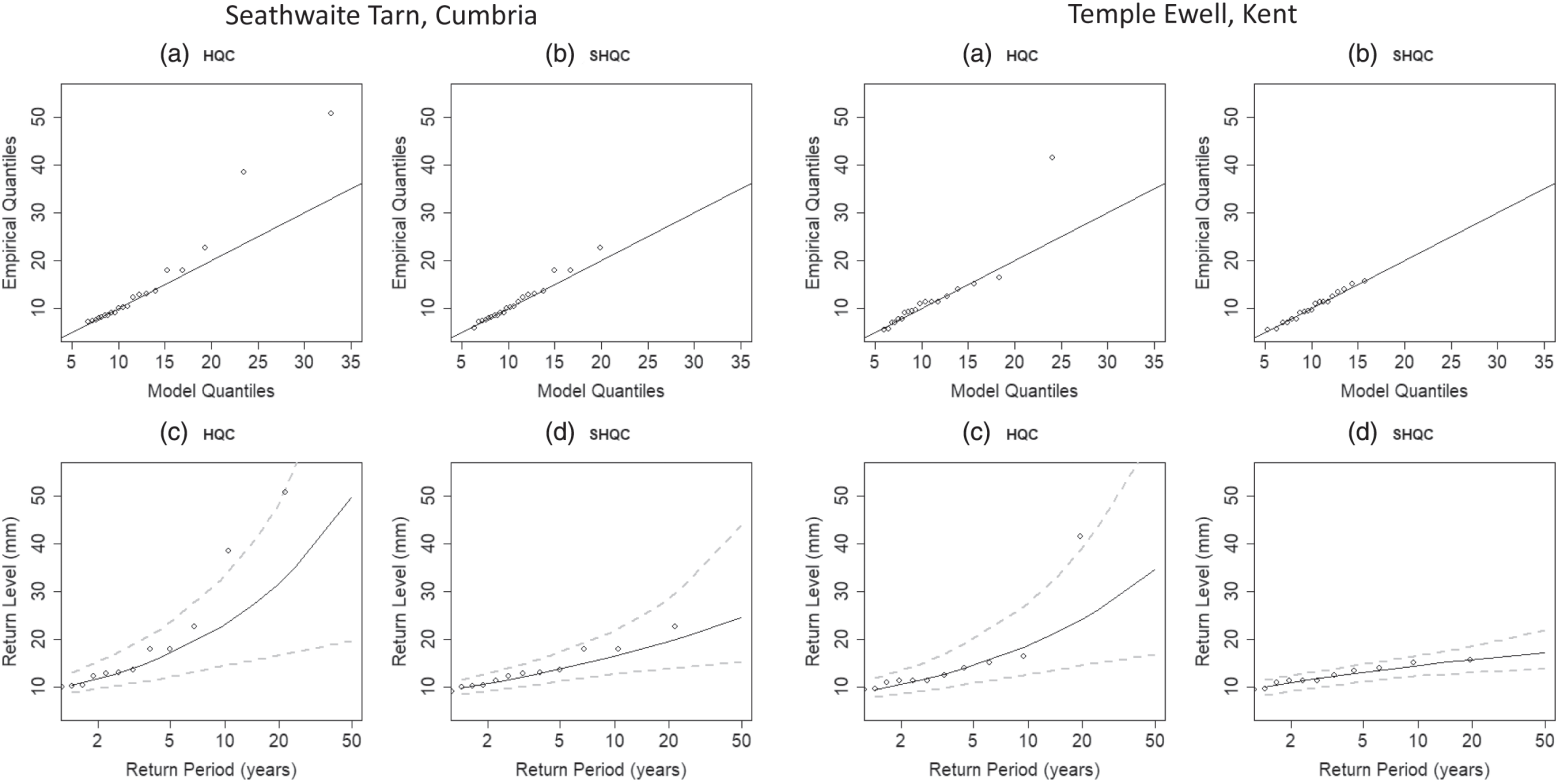
Quantile–quantile plots for the 15 min AMS after (a) HQC and (b) SHQC, with observed quantiles (*y*‐axis) against GEV model quantiles (*x*‐axis) plotted with a 1:1 straight line for reference. GEV return level estimates (solid curves), 95% confidence intervals (dashed lines) and observations (points) for data after (c) HQC and after (d) SHQC are shown for the same gauge and duration. The rain‐gauges shown represent high‐elevation western (Seathwaite Tarn, ∼378 m) and low‐lying eastern (Temple Ewell, ∼29 m) locations

Figure 9 shows how the presence of suspicious large values prior to SHQC produces a poor GEV model fit (panels (a) and (c) for both gauges). Better‐behaved models that more closely approximate the observations are observed after SHQC (Figure 9b,d for both gauges). Note how the GEV models become less convex after SHQC as the estimated ξ is reduced in both gauges.

**FIGURE 10:**
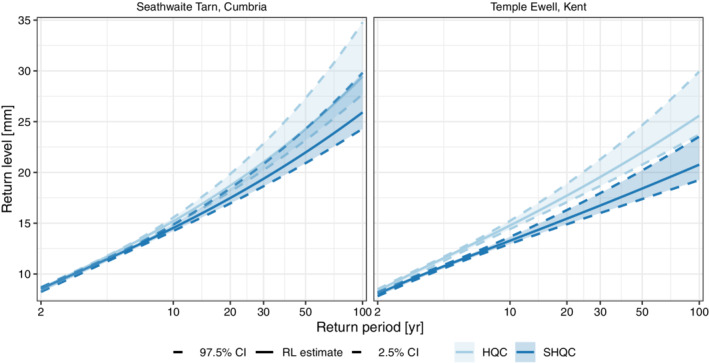
Regional return level plots for 15 min rainfall totals calculated using 30‐gauge regions centred at Seathwaite Tarn and Temple Ewell. Solid lines indicate return level (RL) estimates, whilst dashed lines and shading indicate 95% confidence upper and lower bounds for these estimates [Colour figure can be viewed at wileyonlinelibrary.com]

Regional return level estimates for 15 min accumulations after SHQC are shown to be significantly reduced at both locations – from 29.4 mm to 25.9 mm at Seathwaite Tarn and from 25.6 to 20.8 mm at Temple Ewell – as the SHQC estimates lie outside the confidence intervals of the HQC estimates (Figure 10). The associated confidence intervals are narrower compared to their single‐site estimates, and SHQC is shown to reduce regional ξ values as the curves are less convex after SHQC. In the region around Seathwaite Tarn we also found that SHQC improves the homogeneity of the region, with H reducing from 5.34 (indicating a poor, very heterogeneous region) to 0.55 (acceptably heterogeneous). SHQC worsened the H values in the Temple Ewell region from 0.15 to 0.66, though both indicate acceptable regional heterogeneity.

## 4 CONCLUSIONS

In this article a QC algorithm for rainfall that operates using sub‐hourly data was developed, providing significant improvements to the quality of the resulting dataset, especially when considering applications such as extreme value analysis. The algorithm, based on monthly thresholds and inter‐tip time checks, can identify and remove faults present in sub‐hourly rain‐gauge data whilst accounting for seasonal variations in rainfall intensity. Therefore, the QC algorithm serves as a useful quality assurance tool for rain‐gauge datasets, especially in cases when manually checking all data would be unfeasible and/or for locations where corroborating datasets (e.g. radar or satellite rainfall estimates) are unavailable.

Threshold selection is an important aspect that controls the number of events that are checked and removed. The values we have selected for GB produce good results relative to the manually quality‐controlled training and validation event sets by removing false events whilst retaining known events. However, the thresholds are not perfect and up to 10% of checked events might have been erroneously removed, whilst up to 19% of cleared events might be suspicious, which shows a bias towards preserving suspicious events. We have not observed much sensitivity, in terms of data removed, to changes in threshold values as these are deliberately very high relative to the rainfall intensities observed in GB rain‐gauge records. The SHQC methods can be rapidly tuned to different locations by selecting different TH_60,m_, TH_15,m_ and TH_1,m_ values according to knowledge of local rainfall extremes. An advantage of using a combination of multiple thresholds at different durations is that simple data censorship (as would occur when removing all data above a certain threshold), and its associated risk of removing real extremes, is avoided. Caution should be used when selecting thresholds to balance the removal of faulty data whilst retaining real extremes.

The use of rain‐gauge observations with sub‐hourly resolution (e.g. tip‐times or 15 min totals) provides valuable information for QC that is not available when considering hourly or daily data. The fast‐tip check was confirmed as a good indicator of faults such as snowmelt events or blockages (Upton and Rahimi, 2003) as it can detect very fast changes in rainfall intensity; similar tests are not possible with 15 min data as the aggregation process disguises these changes in intensity. Therefore, rain‐gauge records should be checked using data at the highest resolution available to maximise the information available to evaluate veracity.

The access to high‐frequency data represents a significant barrier to the use of SHQC. In future work it may be possible to use high‐frequency observations to derive indicators of faulty or suspicious observations for use in aggregated data, for example by determining if the signature of snow‐melt faults can be distinguished in hourly data. Machine learning algorithms represent a potentially useful tool for such an analysis.

Extreme rainfall values, such as seasonal maxima and AMS, are improved after SHQC through the removal of spurious values. There is a greater impact on short‐duration rainfall totals (1 hr or shorter) as the aggregation process to obtain 6 or 24 hr totals can mask out the sub‐hourly errors that SHQC evaluates; however, changes were observed across all evaluated accumulation periods (15 min, 1, 3, 6 and 24 hr).

The use of SHQC to remove suspicious data has a significant impact on single‐gauge and regional estimates of the GEV shape parameter from the resulting dataset, with a moderating effect on the distribution tail due to the reduction of large ξ values. ξ is found to decrease with rainfall accumulation period, from 0.14 at 15 min to 0.06 at 24 hr; therefore, extremes of short‐duration rainfall are more heavy‐tailed than extremes of long‐duration rainfall in GB. This also reinforces previous evidence that the types of rainfall event that generate short‐duration extremes differ from those which cause extremes for accumulation periods of 6 hr or greater (Hand *et al*., 2004; Darwish *et al*., 2018).

Conducting SHQC has a significant impact on single‐gauge and regional return level estimates corresponding to 0.2–0.01 AEP events when using the GEV distribution. Differences at individual regions and gauges can be substantial (>10%); however, average differences are under ±2.5% for most regional return level estimates. The reduction in magnitude of the rainfall estimates highlights the importance of QC for engineering applications and to provide improved validation datasets for convection‐permitting climate models.

The work in this article illustrates that different levels of QC may be suitable for different applications. High‐quality QC based on hourly data is sufficient for applications such as water resource management and planning where annual or monthly rainfall volumes are key, as the events removed by additional sub‐hourly QC represent a small contribution to total rainfall volumes. Applications where an accurate description of extreme rainfall is required are those that stand to benefit the most from additional SHQC. These applications include statistical analyses of flooding driven by extreme rainfall such as those witnessed in Europe and China during summer 2021, the development of design rainfall guidance required to provide adequate infrastructure, and the validation of climate model output.

## AUTHOR CONTRIBUTIONS

**Roberto Villalobos Herrera:** Conceptualization; data curation; investigation; methodology; software; writing – original draft. **Stephen Blenkinsop:** Supervision; writing – review and editing. **Selma Guerreiro:** Supervision; writing – review and editing. **Tess O'Hara:** Writing – review and editing. **Hayley Fowler:** Funding acquisition; project administration; supervision; writing – review and editing.

## CONFLICT OF INTEREST

The authors report no conflict of interest.

## CODE AND DATA AVAILABILITY

The GSDR‐QC algorithm is available for download at https://github.com/nclwater/intense‐qc, or https://pypi.org/project/intense/ (Lewis *et al*., 2021). The SHQC scripts are available at https://github.com/nclwater/SubHourlyQC. All scripts are written for Python 3. Licencing agreements with the data providers (EA, NRW and SEPA) prevent us from sharing the quality controlled or original data used in this article. These data are available on request from these institutions. ETCCDI index data are available for download from https://www.climdex.org/learn/datasets/.

## Supporting information


**Appendix S1** Supporting information

## Data Availability

All rainfall data may be requested from the original providers listed in the article. QC code is available, with details in the article.

## Footnotes

1 Great Britain refers to the island where England, Scotland and Wales are located, whilst the UK refers to the country which includes Northern Ireland in addition to Great Britain.
