# Supplementary material for: Sub‐hourly resolution quality control of rain‐gauge data significantly improves regional sub‐daily return level estimates
Source: Q J R Meteorol Soc. 2022 Sep 9;148(748):3252–71. doi: 10.1002/qj.4357 (PMC9826504; doi:10.1002/qj.4357)
Supplement: Supplementary file 1 — Appendix S1 Supporting information [file QJ-148-3252-s001.docx]

# Supplementary information

### Hourly QC

The full GSDR-QC algorithm is comprised of station metadata tests, 25 time-series quality tests which flag potential errors in the rainfall data, and a rule-base which uses the test flags to determine if data is suspect or not. This algorithm uses three datasets: the *hourly* rainfall data subject to QC, a selection of the reference *rainfall indices* defined by the Expert Team on Climate Change Detection and Indices (ETCCDI) as calculated by the Climdex project (Donat, Alexander, Yang, Durre, Vose, & Caesar, 2013; Donat, Alexander, Yang, Durre, Vose, Dunn, et al., 2013), and manually quality-controlled *daily* rainfall data from the Global Precipitation Climatology Centre (GPCC) operated by DWD under the auspices of the WMO (Schneider et al., 2018). The UK rainfall dataset included in the GSDR is different from that used in this study as it contains ~1,900 rain gauges with data up to 2015; the 1,301 gauges used in our study are the subset of the GSDR gauges for which sub-hourly precipitation was available. The gridded ETCCDI indices were calculated using HadEX2 (Donat, Alexander, Yang, Durre, Vose, Dunn, et al., 2013) and GHCNDEX (Donat, Alexander, Yang, Durre, Vose, & Caesar, 2013) *daily* data. A key difference between the indices and daily rainfall reference datasets is that while the ETCCDI datasets are available for download, the GPCC dataset is protected by Third Party agreements (Schneider et al., 2018) and cannot be accessed outside of DWD.

The metadata tests in GSDR-QC are preliminary, manual procedures, and include examining records for erroneous coordinates (for example mapping gauges at sea) or duplicate gauges.

The time-series tests cover all the categories of tests recommended by existing and draft WMO guidelines (WMO, 2018; Chambers et al., 2018), except for the internal consistency tests which rely on multi-instrument data which is beyond the scope of this work, and the spike tests which rely on sub-hourly data to be effective. The breakdown of the GSDR-QC time-series tests according to the WMO’s classification system is shown in Table S1 along with a breakdown of the QC tests included in both the full GSDR-QC and the UK-specific HQC and SHQC algorithms. For a full description of the GSDR-QC tests please refer to (Lewis et al., 2021).

The GSDR-QC algorithm can be modified for ‘local’ use by changing the default parameters and reference datasets. This is described in Lewis et al. (2021), where the GSDR-QC was modified to quality-control an hourly dataset of UK precipitation, resulting in the HQC algorithm used here. The main difference between our HQC and the GSDR-QC is the exclusion of the GPCC dataset due to access restrictions which impede its use outside of DWD. The GSDR-QC daily and monthly wet/dry neighbour tests for spatial consistency of dry and wet periods operate using GPCC daily rainfall dataset and are therefore disabled in HQC. The hourly wet/dry neighbour checks operate using gauges within the rainfall dataset; therefore, spatial consistency tests are retained by HQC. The ETCCDI reference datasets used by GSDR-QC are unchanged for HQC, they are the only external data required by our QC methods and they are available as global gridded datasets (Donat, Alexander, Yang, Durre, Vose, & Caesar, 2013; Donat, Alexander, Yang, Durre, Vose, Dunn, et al., 2013).

Three further modifications were made to GSDR-QC to better reflect UK rainfall characteristics. The ‘world record’ 1-hr rainfall value was adjusted down from 401 mm to the UKMO 1-hr rainfall record of 92 mm and the location-dependent Rx1day ETCCDI parameter was substituted by the UKMO 24-hour rainfall record of 341.4 mm (Met Office, 2020), both were done to better reflect the characteristics of British rainfall. The Rx1day test was then modified to evaluate 24-hour rolling window totals against the UKMO record to make better use of the available information provided by the UKMO record; the original GSDR test evaluates each hourly value against the local Rx1day index. Finally, the streaks check was modified by setting its minimum hourly threshold to a fixed value of 20 mm as lower thresholds removed portions of known large events such as the 4-5 December 2015 24-hour record event at Honister Pass during Storm Desmond.


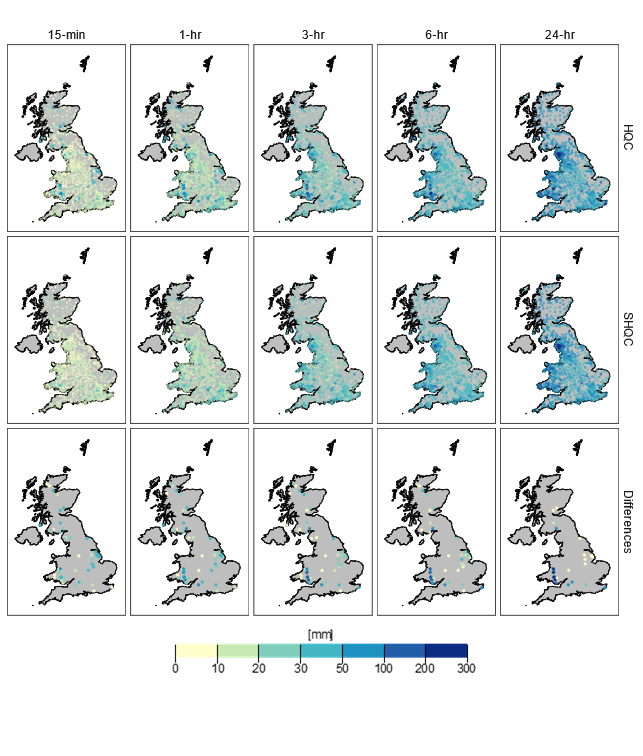


Figure S1. Maximum autumn (September, October, November) rainfall totals [mm] for 15-min, 1-hr, 6-hr and 24-hr accumulation periods, after HQC (top row) and after SHQC (middle row), with differences in magnitude (HQC -SHQC) shown in the final row.


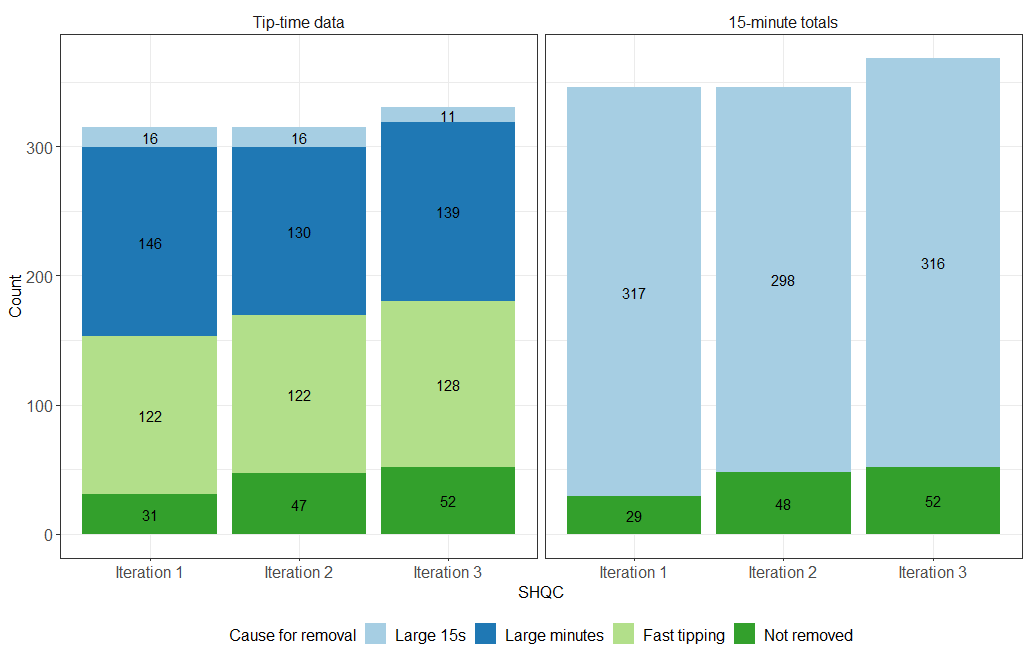


Figure S2. Cause for event removal according to data resolution and SHQC/T iteration.


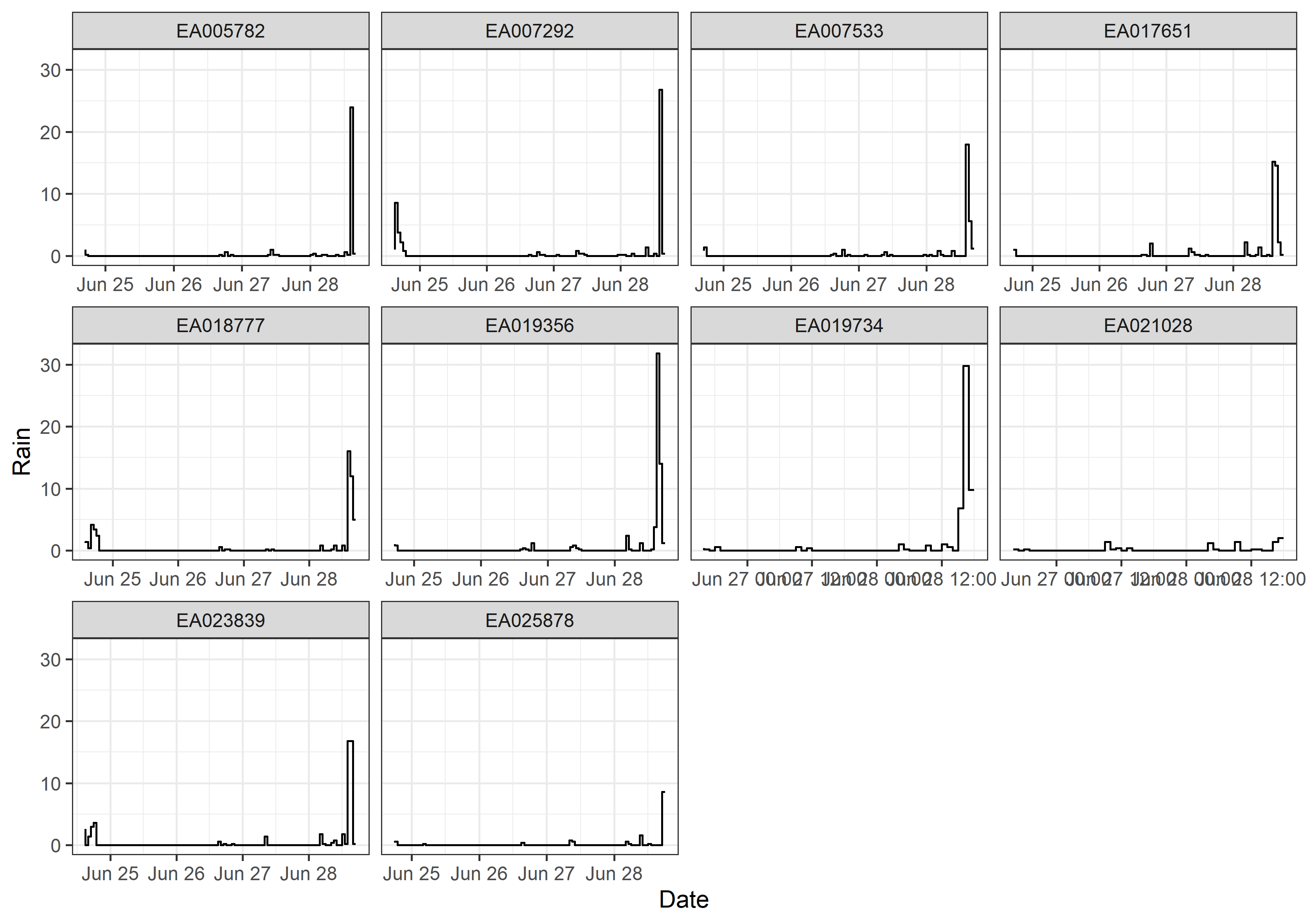


Figure S3. Hourly timeseries plots of the 10 closest gauges to the Newcastle upon Tyne pluvial flood event of 27/06/2012, ordered by increasing distance.


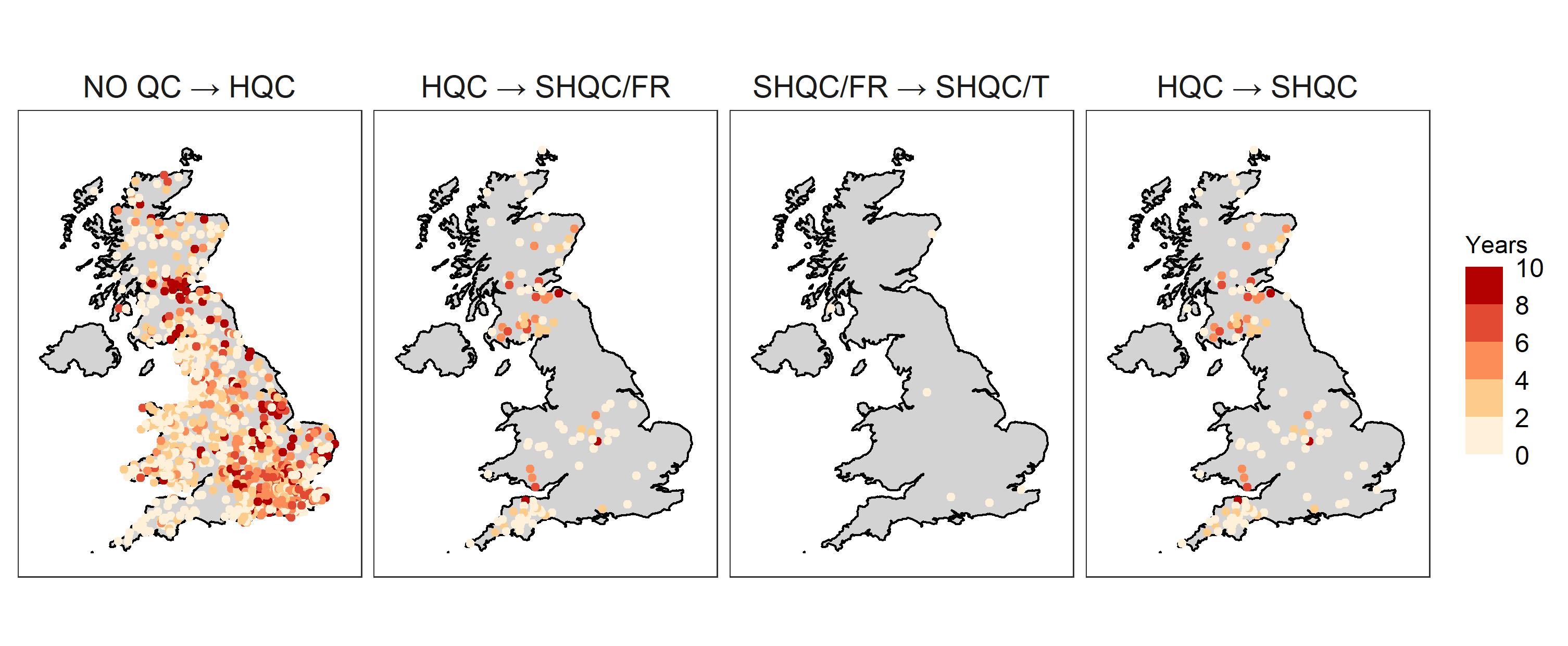


Figure S4. Reduction in gauge AMS length (years) as calculated by subtracting the number of years present in the AMS series of each rain gauge at the QC stages shown in the panel headers. Rain gauges without differences have been omitted. A maximum of 15% missing data is allowed in a year before it is considered incomplete and removed from a gauge’s AMS, therefore the number of years at each QC stage equals the number of complete years in the gauge’s record minus the number of incomplete years. HQC → SHQC represents the combined effect of SHQC/FR and SHQC/T. not an additional QC stage.


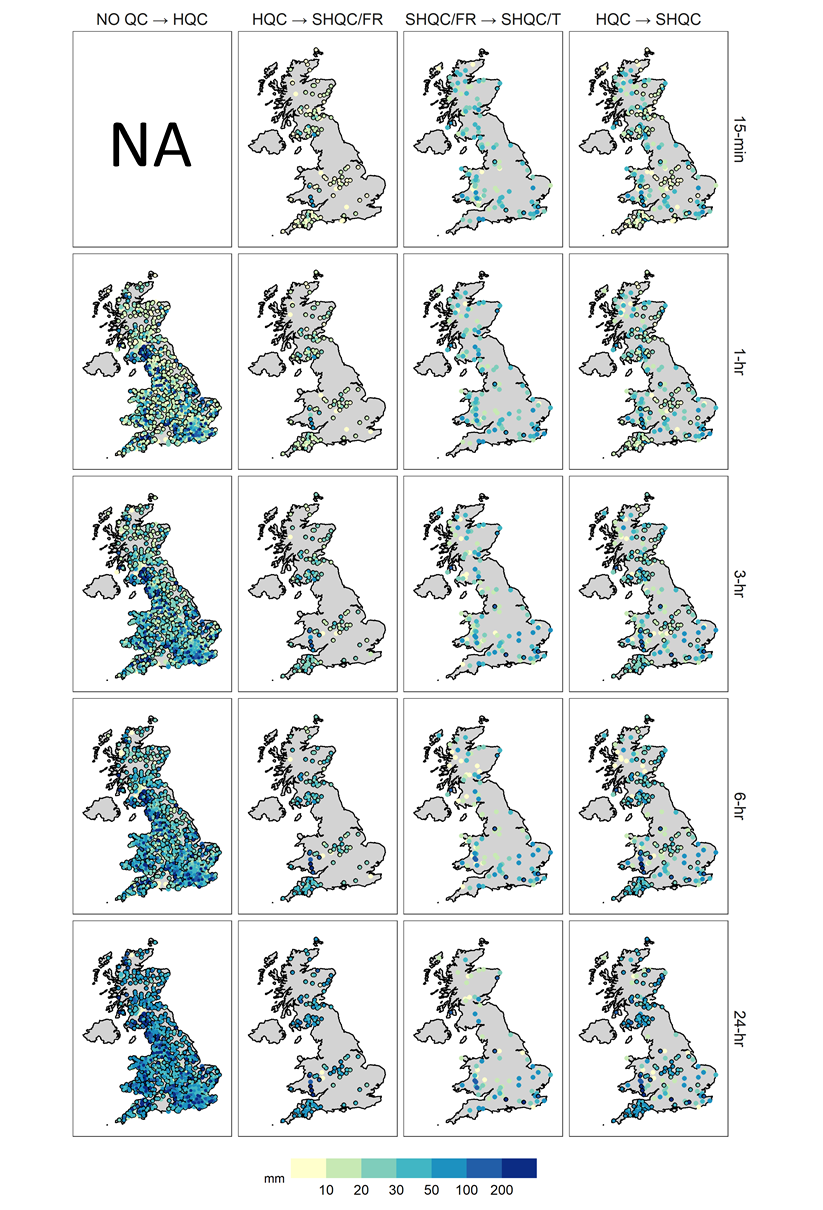


Figure S5. Largest decrease in 15-min, 1-hr, 3-hr, 6-hr, and 24-hr rainfall totals within each rain gauge’s AMS caused by each SHQC stage. Gauges where the length of the AMS has been shortened have a black outline. Differences were calculated between the QC stages shown on the panel headers, HQC → SHQC represents the combined effect of SHQC/FR and SHQC/T, not an additional QC stage. Rain gauges without differences have been omitted.


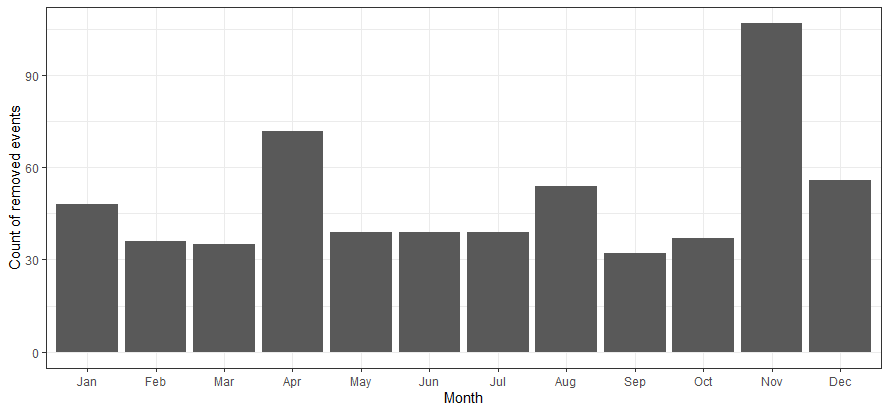


Figure S6. Monthly histogram of events removed by SHQC/T.


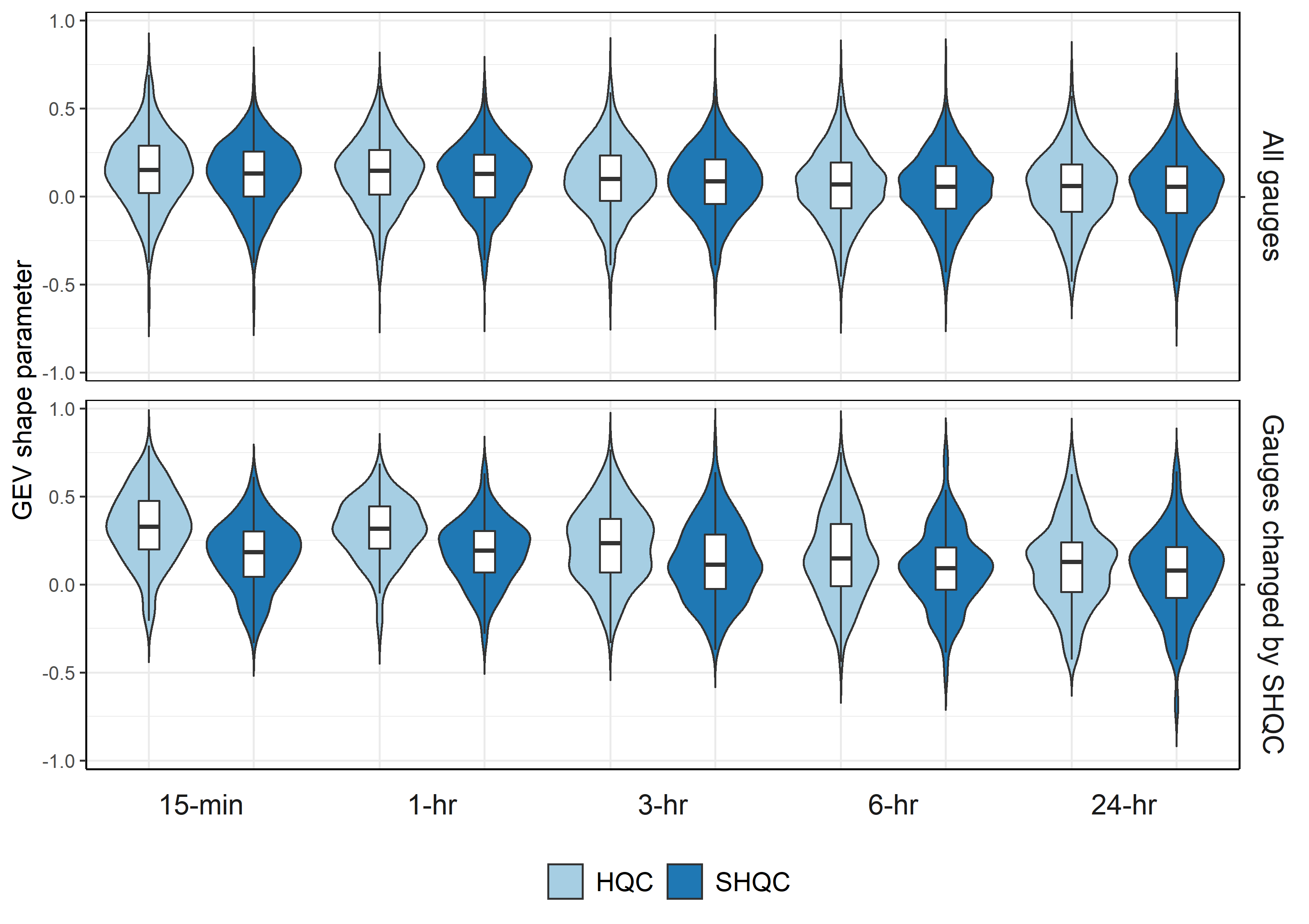


Figure S7. GEV shape parameter violin and box plots at different event accumulation periods from 15-min to 24-hr, after HQC (light fill) and SHQC (dark fill) for all gauges (top) and for gauges where SHQC has removed suspicious data (bottom). Violin plots are density plots, mirrored about the vertical axis and plotted using a kernel density estimate. The bold line in the boxplot inserts shows the median, the upper and lower edges of the box show the 75% and 25% quantiles respectively, whiskers extend up to 1.5 $\times$ the inter-quartile range beyond the box edges.


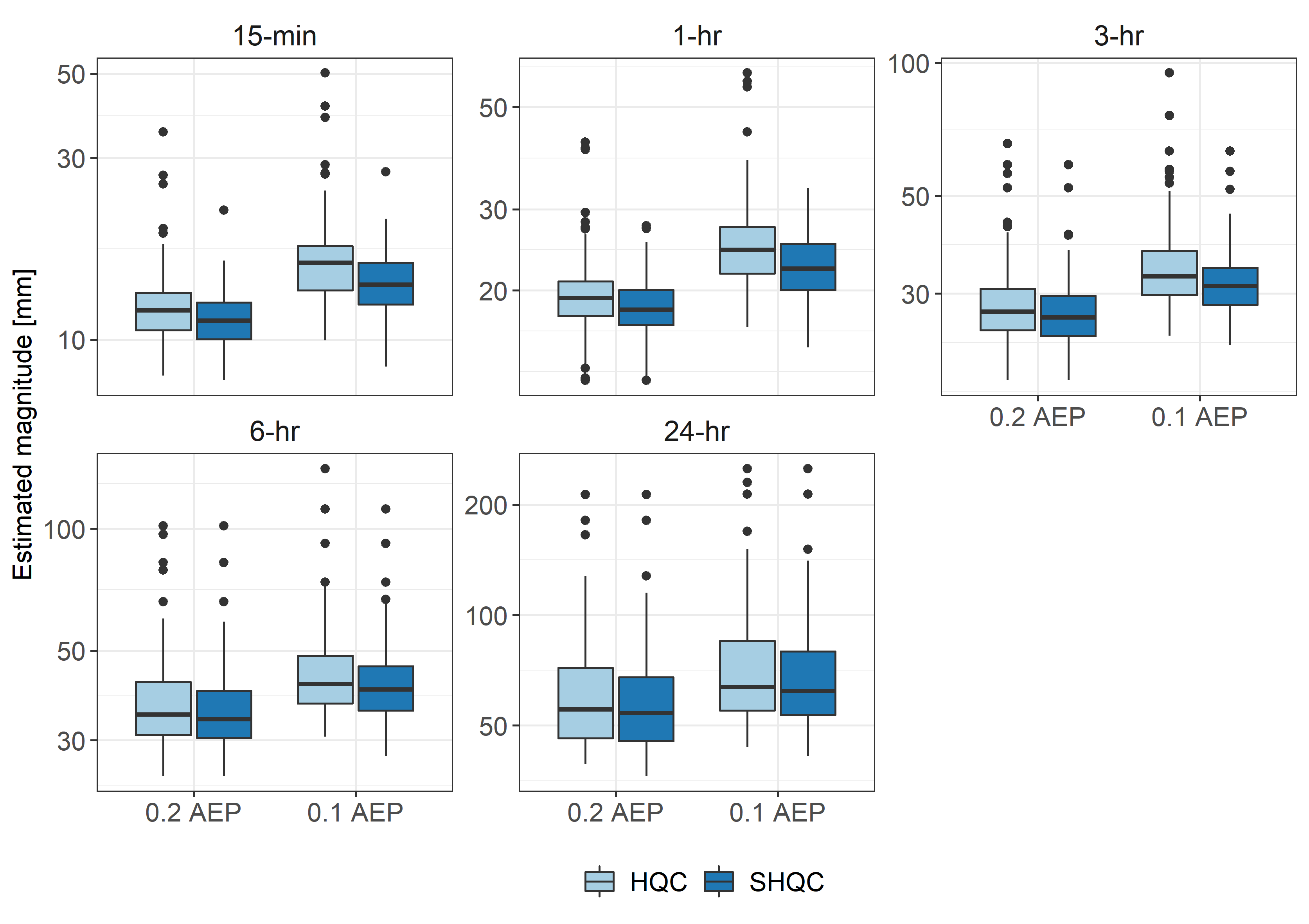


Figure S8 Box plots of return level estimates at 0.2 and 0.1 AEP, after HQC and SHQC, for gauges where SHQC has removed data. The bold line shows the median, the upper and lower edges of the box show the 75% and 25% quantiles respectively, whiskers extend up to 1.5 $\times$ the inter-quartile range beyond the box edges and dots indicate outlying observations.


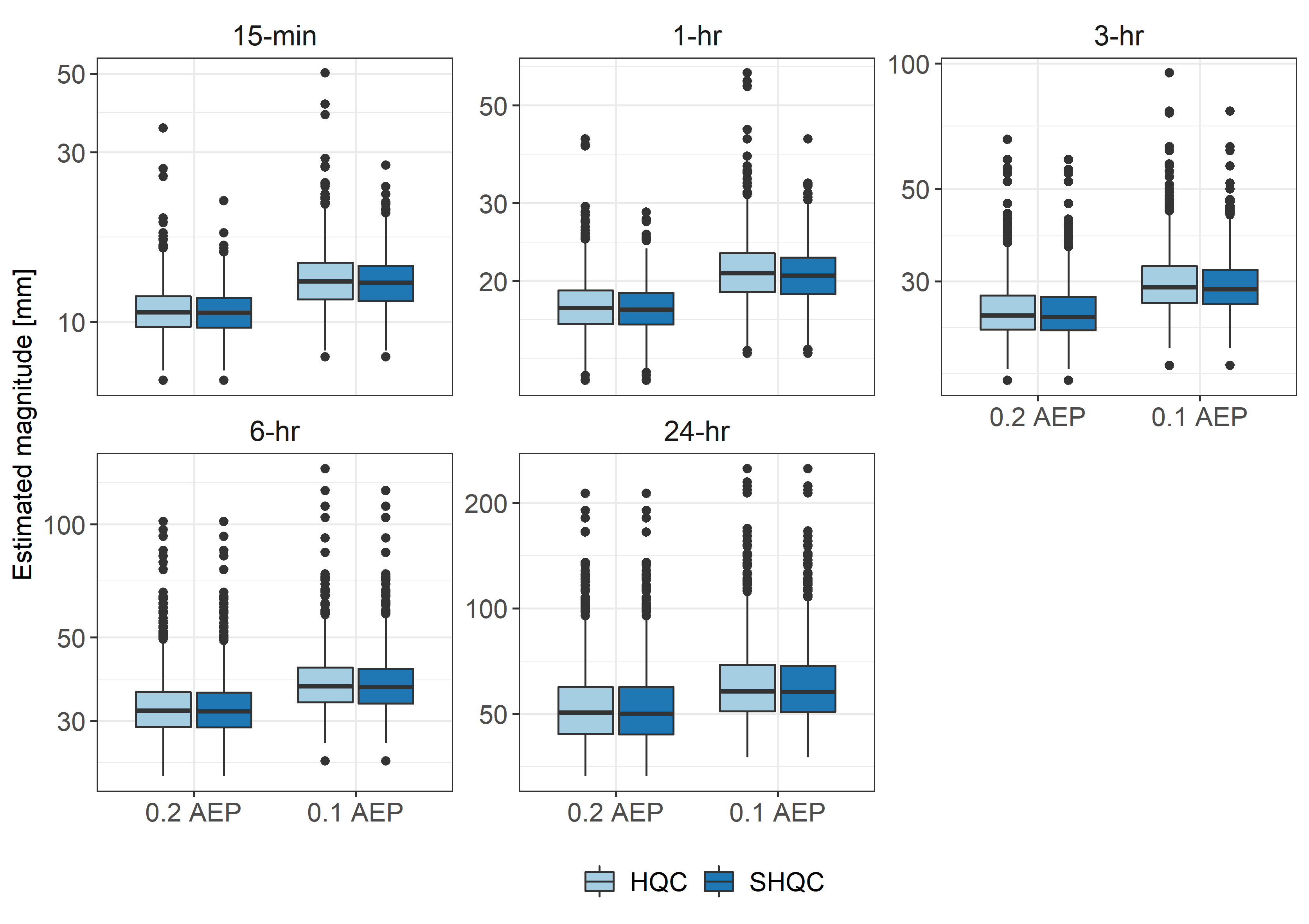


Figure S9. Box plots of return level estimates at 0.2 and 0.1 AEP, after HQC and SHQC, for all gauges. The bold line shows the median, the upper and lower edges of the box show the 75% and 25% quantiles respectively, whiskers extend up to 1.5 $\times$ the inter-quartile range beyond the box edges and dots indicate outlying observations.


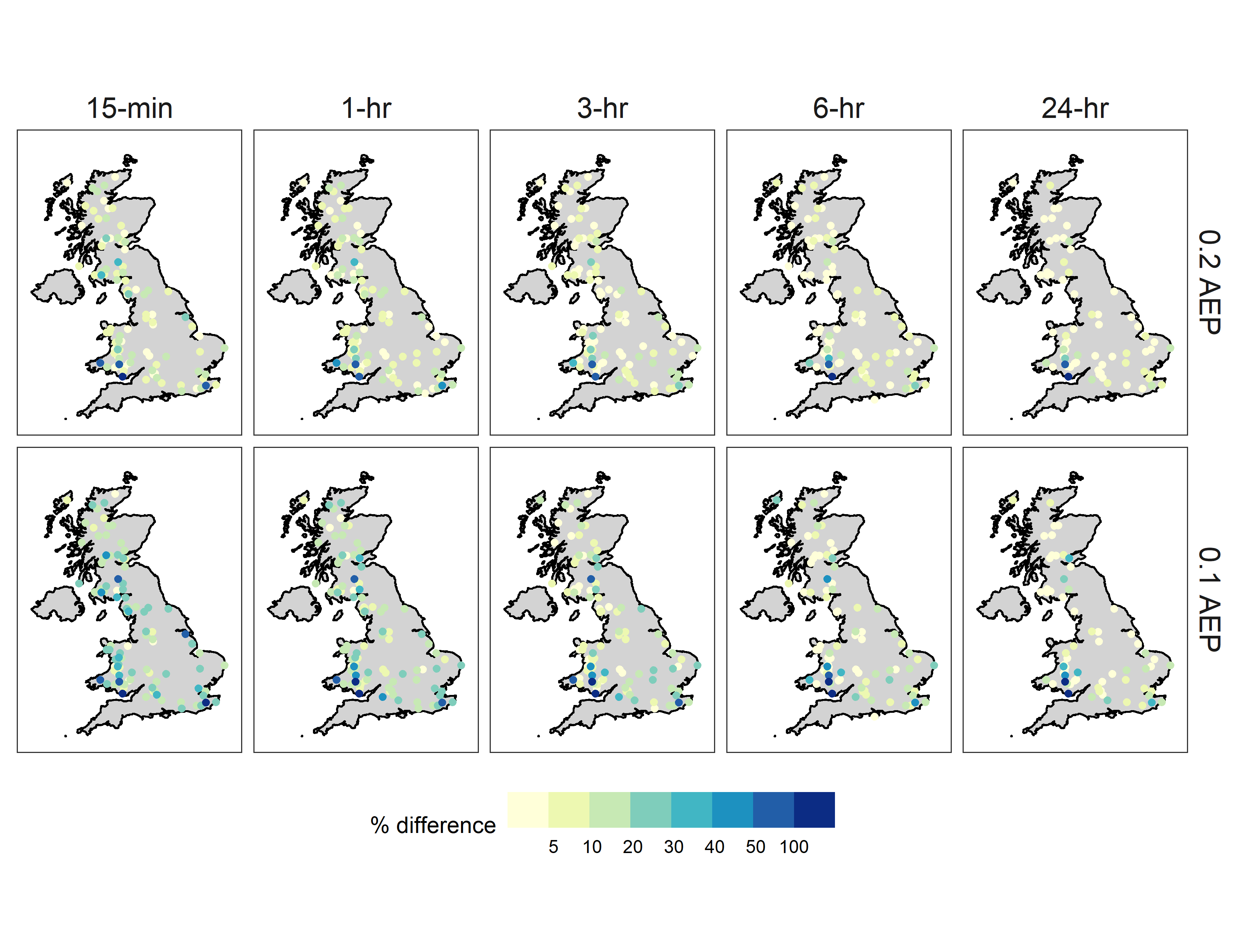


Figure S10. Percentage difference in magnitude estimates before and after SHQC for 0.2 (top row) and 0.1 AEP (bottom row) rainfall events with 15-min,1-hr, 3-hr, and 24-hr accumulation periods, for gauges which have been modified by SHQC/T.

Table S1. QC tests and their classification

i PRCPTOT is the annual total precipitation on days with over 1mm of rainfall (Donat, Alexander, Yang, Durre, Vose, & Caesar, 2013)

ii R99pTOT is the contribution to total rainfall from days exceeding the 99th percentile threshold of all days with over 1mm of rainfall (Donat, Alexander, Yang, Durre, Vose, & Caesar, 2013)

Table S2. Date, location, and maximum 15-min and 1-hr rainfall totals for reference pluvial flood events.

|  |  |  |  |  |  | **Maximum rainfall at a nearby gauge [mm]** | |
| --- | --- | --- | --- | --- | --- | --- | --- |
| **Event #** | **Date** | **Grid Reference** | **Postcode** | **Address (near)** | **Location** | **15-min** | **1-hr** |
| 1 | 18-8-2011 | SY 75800 99754 | Undefined | Cheselbourne | Dorset | 10.4 | 10.4 |
| 2 | 18-8-2011 | SZ 08801 91268 | Undefined | Bournemouth | Bournemouth | 12.2 | 25.0 |
| 3 | 22-6-2012 | SD 47513 61866 | Undefined | Lancaster | Lancaster | 3.0 | 7.4 |
| 4 | 5-8-2012 | SX 96093 76586 | EX7 9FD | Dawlish | Devon | 17.0 | 48.0 |
| 5 | 5-8-2012 | SU 11571 83577 | Undefined | Chudleigh | Swindon | 5.8 | 9.4 |
| 6 | 5-8-2012 | TQ 92526 85927 | Undefined | Kingsteignton | Shoeburyness | 10.6 | 11.8 |
| 7 | 24-9-2012 | SD 47513 61866 | Undefined | Lancaster | Lancaster | 3.0 | 8.2 |
| 8 | 24-9-2012 | SY 96013 92928 | BH16 6JQ | Lytchett Minster | Dorset | 5.2 | 13.8 |
| 9 | 23-7-2013 | SK 61386 49221 | Undefined | Calverton | Nottinghamshire | 20.0 | 37.2 |
| 10 | 23-7-2013 | SK 67318 45927 | NG14 7HH | Lowdham | Nottinghamshire | 20.0 | 37.2 |
| 11 | 20-7-2014 | TQ 78008 84090 | Undefined | Canvey Island | Essex | 18.2 | 38.6 |
| 12 | 8-8-2014 | TL 44801 58606 | CB2 1TB | Bar Hill | Cambridge | 18.8 | 44.2 |
| 13 | 19-9-2014 | TL 85278 64339 | IP33 1BZ | Bury St Edmunds | Suffolk | 3.0 | 4.4 |
| 14 | 4-7-2015 | TL 21199 35988 | SG5 4PS | Stotfold | Bedfordshire | 9.8 | 16.6 |
| 15 | 3-1-2016 | NO 10998 25336 | PH1 | North Muirton | Perth | 1.4 | 2.6 |
| 16 | 4-1-2016 | NO 10998 25336 | PH1 | North Muirton | Perth | 1.2 | 3.0 |
| 17 | 16-6-2016 | SP 05843 82613 | B29 | Pershore Road | Birmingham | 14.6 | 31.2 |
| 18 | 22-8-2016 | SD 28573 78353 | LA12 7DX | South Ulverston | Cumbria | 5.2 | 12.8 |
| 19 | 18-7-2017 | SW 78238 18586 | Undefined | Coverack | Cornwall | 0.8 | 1.6 |
| 20 | 23-8-2017 | TA 04188 88553 | Undefined | Scarborough | Yorkshire | 23.8 | 31.4 |
| 21 | 30-9-2017 | SD 17146 80186 | LA18 5BD | Millom | Cumbria | 5.4 | 14.0 |
| 22 | 21-10-2017 | SD 93641 24142 | Undefined | Todmorden | Yorkshire | 4.6 | 13.2 |
| 23 | 27-5-2018 | SP 83660 48961 | Undefined | Stoke Goldington | Stoke Goldington | 17.6 | 29.6 |
| 24 | 27-5-2018 | SP 85550 39024 | Undefined | Milton Keynes | Milton Keynes | 17.6 | 29.6 |
| 25 | 27-5-2018 | SO 83617 65675 | Undefined | Northampton | Wychavon | 6.6 | 17.6 |
| 26 | 27-5-2018 | SJ 98297 10096 | WS11 0DR | Cannock | Staffordshire | 16.6 | 25.0 |
| 27 | 27-5-2018 | SP 06689 86822 | Undefined | Birmingham, Birmingham | Birmingham | 12.8 | 22.6 |
| 28 | 20-9-2018 | SK 35449 87427 | Undefined | Sheffield, Sheffield | Sheffield | NA | NA |
| 29 | 20-9-2018 | SK 42970 92853 | Undefined | Rotherham, Rotherham | Rotherham | NA | NA |
| 30 | 27-6-2012 | NZ 24531 64319 | NE1 5RZ | Newgate Street | Newcastle upon Tyne | 22.6 | 31.8 |

Table S3. Summary statistics for single-gauge estimates of $\xi$.

| All gauges | | | | | | | | | | |
| --- | --- | --- | --- | --- | --- | --- | --- | --- | --- | --- |
|  | 15-minutes | | 1-hour | | 3-hours | | 6-hours | | 24-hours | |
|  | HQC | SHQC | HQC | SHQC | HQC | SHQC | HQC | SHQC | HQC | SHQC |
| Min | -0.621 | -0.621 | -0.485 | -0.485 | -0.728 | -0.732 | -0.635 | -0.635 | -0.757 | -0.757 |
| Q_5_ | -0.168 | -0.177 | -0.196 | -0.201 | -0.257 | -0.258 | -0.294 | -0.294 | -0.301 | -0.306 |
| Q_25_ | 0.020 | 0.005 | 0.013 | 0.002 | -0.031 | -0.039 | -0.076 | -0.083 | -0.073 | -0.076 |
| Q_50_ | 0.164 | 0.139 | 0.156 | 0.131 | 0.102 | 0.086 | 0.068 | 0.053 | 0.059 | 0.055 |
| Q_75_ | 0.301 | 0.262 | 0.275 | 0.239 | 0.239 | 0.208 | 0.192 | 0.177 | 0.196 | 0.186 |
| Q_95_ | 0.505 | 0.416 | 0.471 | 0.386 | 0.435 | 0.381 | 0.402 | 0.354 | 0.396 | 0.365 |
| Max | 0.795 | 0.605 | 0.714 | 0.613 | 0.775 | 0.775 | 0.736 | 0.736 | 0.711 | 0.691 |
| Mean | 0.159 | 0.127 | 0.145 | 0.117 | 0.102 | 0.079 | 0.061 | 0.044 | 0.054 | 0.046 |
| SD | 0.208 | 0.187 | 0.198 | 0.179 | 0.205 | 0.194 | 0.208 | 0.196 | 0.214 | 0.207 |
|  | | | | | | | | | | |
| Gauges with data removed by SHQC | | | | | | | | | | |
|  | 15-minutes | | 1-hour | | 3-hours | | 6-hours | | 24-hours | |
|  | HQC | SHQC | HQC | SHQC | HQC | SHQC | HQC | SHQC | HQC | SHQC |
| Min | -0.239 | -0.414 | -0.082 | -0.479 | -0.344 | -0.732 | -0.517 | -0.471 | -0.403 | -0.652 |
| Q_5_ | 0.011 | -0.173 | 0.024 | -0.079 | -0.068 | -0.202 | -0.233 | -0.238 | -0.285 | -0.285 |
| Q_25_ | 0.205 | 0.058 | 0.229 | 0.063 | 0.104 | 0.009 | -0.017 | -0.081 | -0.061 | -0.092 |
| Q_50_ | 0.355 | 0.187 | 0.352 | 0.184 | 0.265 | 0.135 | 0.175 | 0.064 | 0.107 | 0.071 |
| Q_75_ | 0.499 | 0.298 | 0.459 | 0.313 | 0.403 | 0.258 | 0.345 | 0.193 | 0.319 | 0.228 |
| Q_95_ | 0.671 | 0.450 | 0.575 | 0.432 | 0.566 | 0.474 | 0.573 | 0.456 | 0.558 | 0.435 |
| Max | 0.795 | 0.539 | 0.714 | 0.613 | 0.775 | 0.775 | 0.736 | 0.736 | 0.711 | 0.691 |
| Mean | 0.338 | 0.165 | 0.332 | 0.179 | 0.249 | 0.126 | 0.165 | 0.074 | 0.121 | 0.075 |
| SD | 0.207 | 0.190 | 0.174 | 0.174 | 0.208 | 0.212 | 0.248 | 0.220 | 0.250 | 0.228 |

References

Chambers, L., Flannery, J., Flannery, P., Hechler, P., Spengler, R. & Wright, W. (2018) *WMO Guidelines on Surface Station Data Quality Assurance for Climate Applications (draft)*.

Donat, M.G., Alexander, L. V., Yang, H., Durre, I., Vose, R. & Caesar, J. (2013) Global land-based datasets for monitoring climatic extremes. *Bulletin of the American Meteorological Society*. 94 (7), 997–1006.

Donat, M.G., Alexander, L. V., Yang, H., Durre, I., Vose, R., Dunn, R.J.H., Willett, K.M., Aguilar, E., Brunet, M., Caesar, J., Hewitson, B., Jack, C., Klein Tank, A.M.G., Kruger, A.C., Marengo, J., Peterson, T.C., Renom, M., Oria Rojas, C., Rusticucci, M., et al. (2013) Updated analyses of temperature and precipitation extreme indices since the beginning of the twentieth century: The HadEX2 dataset. *Journal of Geophysical Research Atmospheres*. 118 (5), 2098–2118.

Lewis, E., Pritchard, D., Villalobos-Herrera, R., Blenkinsop, S., McClean, F., Guerreiro, S., Schneider, U., Becker, A., Finger, P., Meyer-Christoffer, A., Rustemeier, E. & Fowler, H.J. (2021) Quality control of a global hourly rainfall dataset. *Environmental Modelling & Software*. 144105169.

Met Office (2020) *UK climate extremes - Rainfall*. [Online]. Available from: https://www.metoffice.gov.uk/research/climate/maps-and-data/uk-climate-extremes (Accessed 17 July 2020).

Schneider, U., Finger, P., Meyer-Christoffer, A., Ziese, M. & Becker, A. (2018) Global Precipitation Analysis Products of the GPCC. Global Precipitation Climatology Centre (GPCC) (June).

WMO (2018) *Guide to climatological practices, second edition.* World Meteorological Organization.
